# Maternal xNorrin, a Canonical Wnt Signaling Agonist and TGF-β Antagonist, Controls Early Neuroectoderm Specification in *Xenopus*

**DOI:** 10.1371/journal.pbio.1001286

**Published:** 2012-03-20

**Authors:** Suhong Xu, Feng Cheng, Juan Liang, Wei Wu, Jian Zhang

**Affiliations:** 1 State Key Laboratory of Molecular Developmental Biology, Institute of Genetics and Developmental Biology, Chinese Academy of Sciences, Beijing, China; 2 Graduate School of Chinese Academy of Sciences, Beijing, China; 3 Protein Science Laboratory of the Ministry of Education, School of Life Sciences, Tsinghua University, Beijing, China; The Wellcome Trust Sanger Institute, United Kingdom

## Abstract

Dorsal–ventral specification in the amphibian embryo is controlled by β-catenin, whose activation in all dorsal cells is dependent on maternal Wnt11. However, it remains unknown whether other maternally secreted factors contribute to β-catenin activation in the dorsal ectoderm. Here, we show that maternal *Xenopus* Norrin (xNorrin) promotes anterior neural tissue formation in ventralized embryos. Conversely, when xNorrin function is inhibited, early canonical Wnt signaling in the dorsal ectoderm and the early expression of the zygotic neural inducers *Chordin*, *Noggin*, and *Xnr3* are severely suppressed, causing the loss of anterior structures. In addition, xNorrin potently inhibits BMP- and Nodal/Activin-related functions through direct binding to the ligands. Moreover, a subset of Norrin mutants identified in humans with Norrie disease retain Wnt activation but show defective inhibition of Nodal/Activin-related signaling in mesoderm induction, suggesting that this disinhibition causes Norrie disease. Thus, xNorrin is an unusual molecule that acts on two major signaling pathways, Wnt and TGF-β, in opposite ways and is essential for early neuroectoderm specification.

## Abstract 2

*Xenopus* maternal Norrin, which activates Wnt signaling but inhibits TGF-β family molecules, is essential for neuroectoderm formation. Loss of TGF-β inhibition in Norrin may contribute to the development of Norrie disease.

## Author Summary

A key step during early embryogenesis is the generation of neural precursors, which later form the central nervous system. In vertebrates, this process requires proper dorsal–ventral axis specification, and we know that the canonical Wnt and BMP signaling pathways help pattern the dorsal ectoderm. In this study, we examine other factors that are involved in neuroectoderm development in the frog species *Xenopus laevis*. We find that maternal *Xenopus* Norrin (xNorrin) is required for canonical Wnt signaling in the dorsal ectoderm, functions upstream of neural inducers, and is required for neural formation. We also find that xNorrin not only activates Wnt signaling, but also inhibits BMP/Nodal-related signaling. In humans, mutations in Norrin cause Norrie disease. Using Norrin mutants identified in patients with Norrie disease, we find that some Norrin mutants fail to inhibit BMP/Nodal-related signaling (specifically, TGF-β) but retain the ability to activate the Wnt pathway, suggesting that loss of TGF-β inhibition may contribute to Norrie disease development.

## Introduction

Dorsal–ventral axis specification is one of the earliest patterning events in the embryo. In vertebrates, early dorsal ectoderm gives rise to the neural plate, which in turn develops into the central nervous system (CNS). Previous studies have found that dorsal axis formation in amphibians is initiated during cortical rotation after fertilization. Current evidence strongly suggests that the canonical Wnt signaling pathway, operating at blastula stages, plays a critical role in dorsal specification [1]. For example, Wnt signaling was discovered to induce secondary axes when ectopically activated in the ventral cells of early embryos. Loss-of-function studies indicate that the Wnt/β-catenin signaling pathway is also essential for dorsal specification [2]–[4]. More recently, Heasman and colleagues provided strong evidence that vegetally localized maternal Wnt11 cooperates with Wnt5A to activate the canonical Wnt pathway and is required for dorsal axis formation [5]–[7]. However, despite extensive studies on dorsal specification, some observations cannot be fully explained. For example, although the cortex is rotated only 30° toward the dorsal side, activated nuclear β-catenin is observed in all dorsal cells, including dorsal cells near the animal pole [8]. Previous studies suggested that Wnt pathway components may be transferred beyond 30° to the dorsal animal region [8],[9]. However, it remains unknown whether such movements can fully account for Wnt activation in dorsal animal cells, and it is also unclear how these movements precisely regulate the earliest steps of neuroectoderm formation in the blastula.

In addition to canonical Wnt signaling, the BMP pathway has also been implicated in neuroectoderm specification and patterning. During early gastrulation, Noggin, Chordin, and Follistatin expressed in the Spemann organizer bind to BMPs in the extracellular space and antagonize their epidermal-promoting effects [10]–[12]. These results support a “default model” for neural induction in which ectoderm cells are predisposed to become neurons if they receive no BMP signals [13],[14]. Genetic screens in *Drosophila* and zebrafish have yielded mutants that affect dorsal–ventral patterning. Interestingly, most of these mutants show defects in the BMP signaling pathway, indicating that BMP signaling has a conserved role in dorsal–ventral patterning [1].

On the other hand, dorsal animal cells in the *Xenopus* blastula can develop into dorsal and neural tissues cell-autonomously when cultured in a saline solution [15],[16]. De Robertis and colleagues found that a subset of the dorsal ectoderm cells in the late blastula expressed *Chordin*, *Noggin*, *Siamois*, and *Xnr3* prior to Spemann organizer functioning and referred to these cells as the blastula *Chordin-* and *Noggin*-expressing center (BCNE center) [16]. Early *Chordin* and *Noggin* transcription is activated by maternal β-catenin, but the precise mechanism underlying this activation remains to be uncovered [16].

We report here that maternal *Xenopus* Norrin (xNorrin) is required for β-catenin activation in dorsal animal cells in the *Xenopus* blastula and in early neuroectoderm development. Norrin is a non-Wnt ligand that was previously shown to activate β-catenin through LRP5 and Frizzled4 or TSPAN12 during retina vascular development [17]–[19]. In humans, mutations in Norrin cause Norrie disease [20]. We further show that xNorrin can directly antagonize TGF-β/BMP signaling. Our results not only identify an endogenous maternal factor required for early neuroectoderm specification, but may also add TGF-β inhibition to the increasingly complex regulatory activities of Norrin in retinal vascular development [17],[19].

## Results

### xNorrin Promotes Dorsal and Anterior Neural Formation

**Figure 1:**
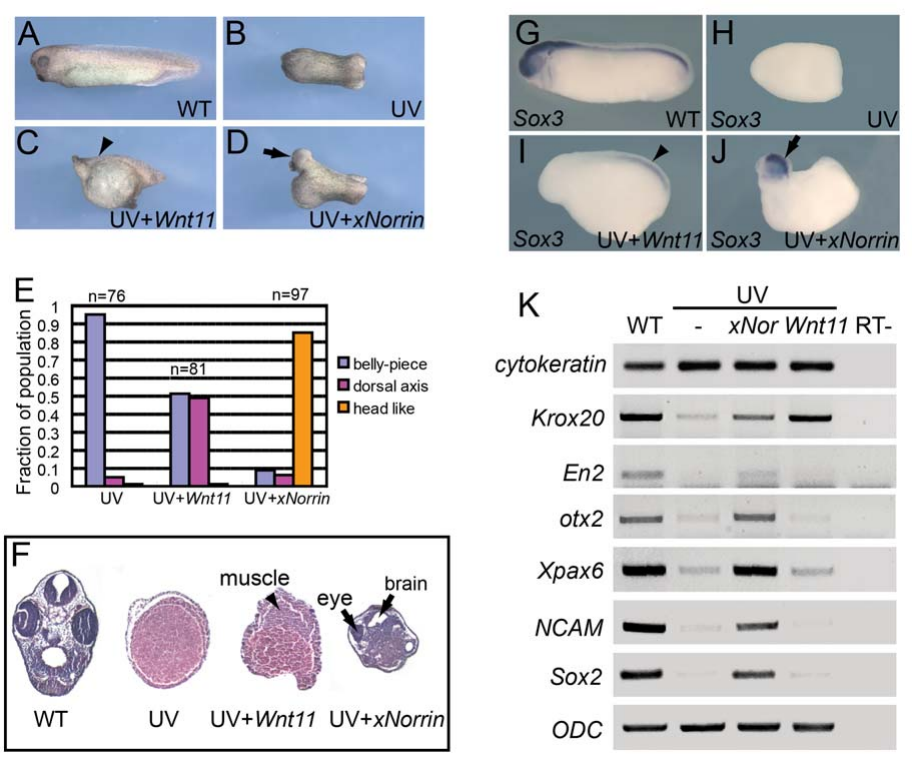
xNorrin induces anterior CNS formation in ventralized embryos. (A–D) *xNorrin* mRNA induces anterior neural tissues, while *Wnt11* mRNA restores only a partial dorsal axis (without anterior structures) in UV-irradiated embryos. (A) A wild-type embryo (stage 33); (B) an embryo UV-irradiated (50 µJ) at the vegetal pole; (C) a UV-irradiated embryo injected with *Wnt11* mRNA (500 pg) into one cell at the four-cell stage (arrowhead: partial dorsal axis); (D) a UV-irradiated embryo injected with *xNorrin* mRNA (500 pg) as in (C) (arrow: head). (E) Summary of (A–D). Fraction of the population within each group is indicated. (F) Histological analysis of stage 40 embryos. Arrowhead: muscle; arrows: brain and eye. (G–J) Whole-mount in situ hybridizations to *Sox3*. (G) A wild-type (WT) embryo (100%, *n* = 65); (H) a UV-treated embryo (4% *Sox3* positive, *n* = 70); (I) a UV+*Wnt11* (500 pg) rescued embryo (45% *Sox3* positive, *n* = 77); (J) a UV+*xNorrin* (500 pg) rescued embryo (83% *Sox3* positive, *n* = 69). All embryos are shown with the anterior pole to the left. Arrowhead: posterior neural structure; arrow: anterior neural structure. (K) Neural marker expression detected by RT-PCR. *xNorrin* induced expression of anterior neural and pan-neural markers (*En2*, *otx2*, *Xpax6*, *NCAM*, and *Sox2*) in UV-irradiated embryos. *Wnt11* induced only the hindbrain marker *Krox20* in UV-irradiated embryos.

We sought to identify additional secreted molecules that are involved in neuroectoderm formation. Neuroectoderm is derived from dorsal animal regions in early *Xenopus* embryos. Therefore, we used early neural markers to search for molecules that may be responsible for early neural specification. In *Xenopus*, ultraviolet (UV) irradiation of the vegetal pole in embryos causes severe dorsal axis development defects [21] (Figure 1A–1F) in otherwise normal embryos (Figure 1A). We selected a set of candidate genes that were previously shown to activate Wnt/β-catenin pathways and tested their ability to reorganize the dorsal axis or anterior neural tissues by injecting them individually into UV-irradiated embryos. Among the maternally expressed *Wnt* genes (*Wnt5a*, *Wnt8b*, and *Wnt11*) tested, *Wnt11* and *Wnt8b* were able to induce some dorsal axis structures (Figure 1C and data not shown) [22],[23]. However, none of these molecules triggered the formation of anterior neural tissues (data not shown).

We also cloned *X. laevis xNorrin* (GenBank accession number: EU528658) from unfertilized eggs. This gene encodes a homolog of human Norrin that can activate β-catenin [18]. The injection of *xNorrin* mRNA into UV-ventralized embryos produced a well-defined head-like structure (Figure 1), including cement gland, eye, and brain-like tissues (85%, *n* = 97). In contrast, only 5% of UV-ventralized embryos (*n* = 76) developed any dorsal axial structures (such as notochord and neural tube), and 49% of *Wnt11*-injected, UV-irradiated embryos (*n* = 81) developed dorsal ridges without notochords and neural tubes (Figure 1C and 1F). Gene expression analysis showed that *xNorrin* induced not only pan-neural markers, such as *Sox3*, *Sox2*, and *NCAM*, but also anterior neural markers, such as *otx2*, *Xpax6*, and *En2*, in stage 20 embryos (Figure 1J and 1K). In contrast, *Wnt11* induced the expression of the rhombomere marker *Krox20* (Figure 1K) and only weakly induced the expression of the pan-neural marker *Sox3* (Figure 1I). These results indicate that *xNorrin* can promote anterior neural tissue formation in an otherwise non-neural background. The neural formation triggered by xNorrin expression in UV-ventralized embryos may perhaps be attributable to early neuroectoderm induction by the injected xNorrin.

**Figure 2:**
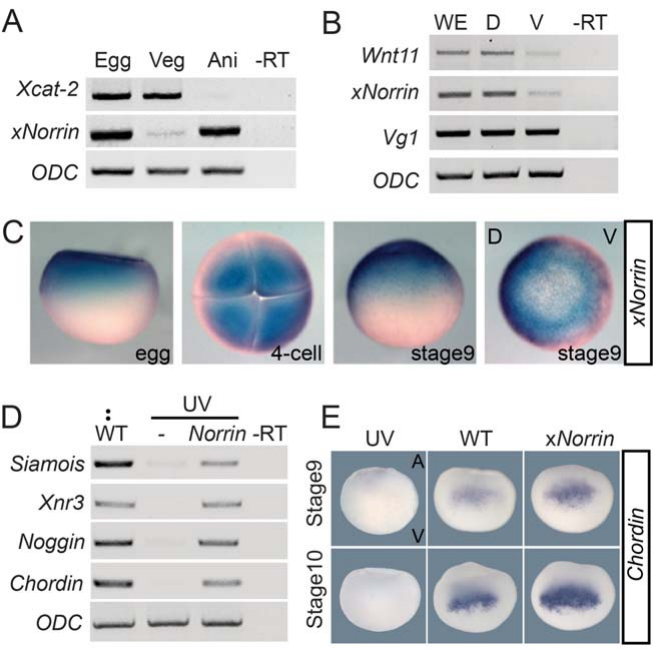
Maternal xNorrin activates the canonical Wnt signaling pathway. (A) RT-PCR analysis of mRNAs from equatorially bisected oocytes (Egg). x*Norrin* mRNA is present in the animal half (Ani) of fully grown oocytes, while *Xcat-2* mRNA is present in the vegetal half (Veg). –RT: no reverse transcription. (B) Both *xNorrin* mRNA and *Wnt11* mRNA are enriched in the dorsal cells of 16-cell embryos. Embryos are evenly bisected into dorsal and ventral halves. D, dorsal half; V, ventral half; WE, whole embryo. (C) Spatial and temporal expression patterns of *xNorrin* mRNA from fertilized eggs to the late blastula stage (stage 9) revealed by whole-mount in situ hybridization. (D) *xNorrin* mRNA (500 pg) injection into the animal region of UV-ventralized embryos at one-cell stage reactivates the expression of *Siamois*, *Chordin*, *Noggin*, and *Xnr3* at the late blastula stage. (E) *xNorrin* injection enhanced *Chordin* expression (detected by in situ hybridization) at stage 9 (81%, *n* = 36) and stage 10 (80%, *n* = 35) compared to wild-type embryos. UV, UV-irradiated embryos; WT, wild-type embryos; xNorrin, wild-type embryos injected with *xNorrin* (500 pg) into the dorsal-animal region at the four- to eight-cell stage.

We reasoned that for maternal xNorrin to act in specifying the neuroectoderm in a cell-autonomous fashion, it should meet two criteria. First, it should be expressed in the dorsal ectoderm of the blastula [16],[24]. Second, it should be able to activate canonical Wnt signaling [16],[25]. Indeed, we confirmed that *xNorrin* mRNA is expressed in the animal pole of stage 6 oocytes and early cleavage embryos (Figures 2A, 2C, and S2A). In addition, much more *xNorrin* mRNA was detected in dorsal blastomeres than in ventral blastomeres in 16-cell-stage embryos (Figure 2B).

Norrin proteins are highly conserved among vertebrates (Figure S1A and S1B). xNorrin, like its mouse ortholog, can activate Wnt-responsive reporters (data not shown) and induce LRP6 phosphorylation in HEK293T cells (Figure S2C). Next, we examined whether *xNorrin* could activate early Wnt target gene expression in vivo. The injection of *xNorrin* into UV-irradiated embryos robustly induced the expression of the known Wnt targets *Chordin*, *Noggin*, *Xnr3*, and *Siamois* (Figure 2D). Further, animal caps injected with *xNorrin* plus *Xenopus Frizzled4* plus human *Lrp5* mRNA (*NFL*) also expressed *Xnr3* and *Siamois*, but not *Xbra* (Figure S2B). We noted that *xNorrin* injection alone did not induce *Xnr3* or *Siamois* expression in animal caps (Figure S2B), suggesting that some components of the xNorrin pathway may not be expressed in the caps (see Discussion). However, the injection of *xNorrin* into dorsal animal cells enhanced *Chordin* expression during the late blastula and early gastrula stages (Figure 2E). These results suggest that maternal *xNorrin* may promote neuroectoderm specification by activating canonical Wnt signaling.

### xNorrin Is Required for Neuroectoderm Specification

**Figure 3:**
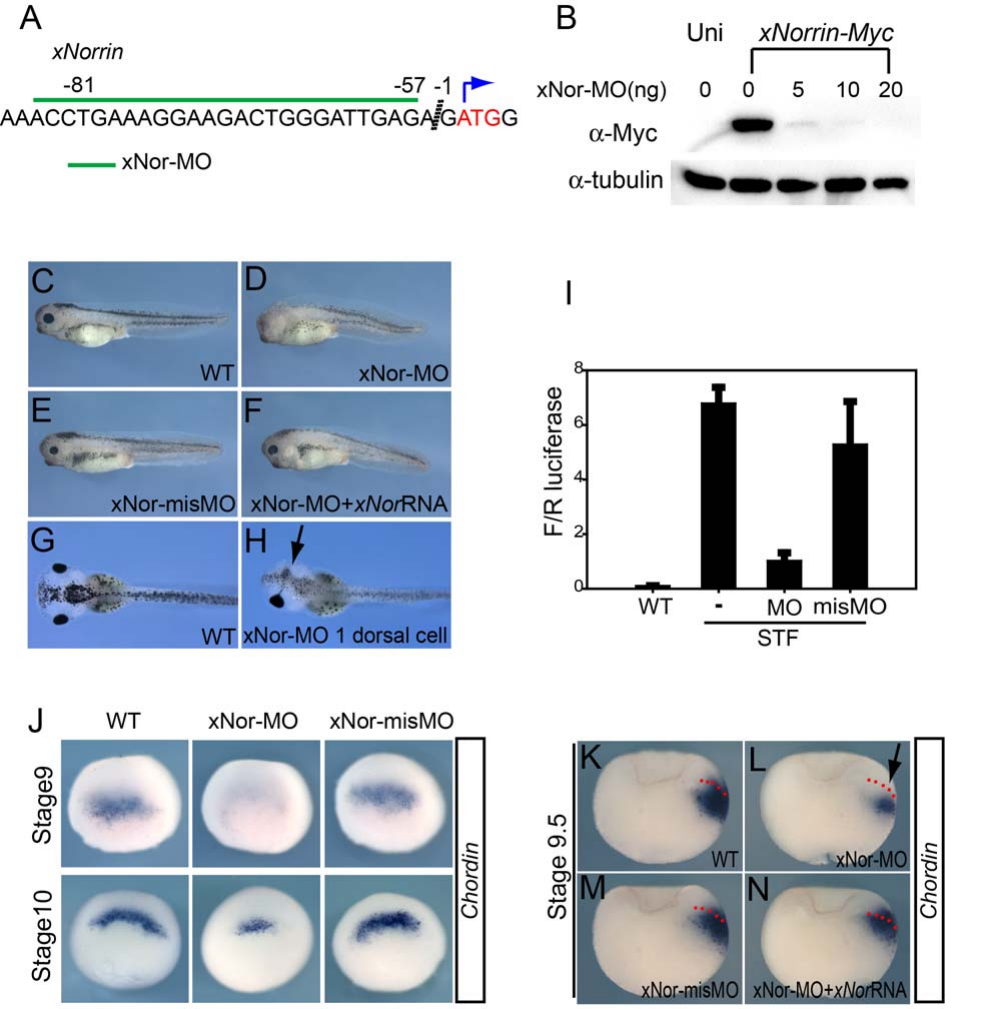
Maternal xNorrin is required for dorsal ectoderm specification. (A) The xNor-MO target sequence (green line) in *xNorrin* mRNA. (B) xNor-MO dose-dependently suppresses *xNorrin-Myc* (1.5 ng) mRNA translation in *Xenopus* embryos. xNorrin-Myc was detected using an anti-c-Myc monoclonal antibody. Uni, no *xNorrin-Myc* injected. (C–F) xNorrin is required for head formation. (C) A wild-type (WT) stage 35 tadpole. (D) xNor-MO (20 ng) caused anterior truncation (61%, *n* = 64) when injected into the animal regions of two dorsal cells in four- to eight-cell-stage embryos. (E) xNor-misMO-injected embryos are generally normal (88%, *n* = 66). (F) The anterior defects caused by NorMO were rescued by *xNorrin* (25 pg) mRNA (77%, *n* = 81). (G) Dorsal view of a wild-type tadpole at stage 45. (H) Anterior defects on only one side (arrow) were generated by injecting xNor-MO (10 ng) into one dorsal cell at the four- to eight-cell stage (63% of injected embryos showed defects in the injected side, *n* = 30). The other side shows normal morphology. The anterior end is to the left. (I) xNor-MO inhibits Wnt signaling in dorsal animal cells. xNor-MO and SuperTopFlash (STF) reporter plasmids were co-injected into the dorsal animal cells of eight-cell embryos. F/R luciferase: ratio of firefly luciferase reading to renilla luciferase reading. (J) Whole-mount in situ hybridization shows that *Chordin* expression is reduced at stage 9 (53% of injected embryos, *n* = 80) and stage 10 (61% of injected embryos, *n* = 79) in xNor-MO-injected embryos, compared to xNor-misMO-injected embryos or uninjected embryos. (K–N) Whole-mount in situ hybridization for *Chordin* in bisected xNor-MO-injected embryos (stage 9.5) showing that xNor-MO inhibits *Chordin* expression in neuroectoderm precursors (arrow) (reduction in 66% of injected embryos, *n* = 104) (L) compared to wild-type embryos (K) and embryos with xNor-misMO injected into dorsal animal cells at the eight- to 16-cell stage (reduction in 13% of injected embryos, *n* = 78) (M). Note that *xNorrin* mRNA (100 pg) rescues *Chordin* expression in the dorsal ectoderm (80% of co-injected embryos showed expression comparable to wild-type embryos, *n* = 50) (N). Embryos are oriented such that their dorsal side is on the right. Dotted lines indicate the boundaries between the deep mesoderm and the superficial ectoderm.

To address whether maternal *xNorrin* is required for neuroectoderm specification and hence anterior CNS formation at a later stage, we used an *xNorrin* antisense morpholino (MO) oligonucleotide (xNor-MO) to inhibit *xNorrin* translation (Figure 3A). The inhibition of *xNorrin* mRNA translation by xNor-MO was both specific and dose-dependent (Figure 3B). We injected xNor-MO into the animal region of the two dorsal blastomeres in the four- to eight-cell embryo stage to suppress endogenous *xNorrin* translation. The majority of xNor-MO-injected embryos (61%, *n* = 64) displayed anterior head truncations, and another 15% of the embryos lacked morphological eye structures and other anterior neural structures at tadpole stages (Figure 3D). The injection of a mismatched MO (misMO), xNor-misMO, that failed to block *xNorrin-Myc* translation (data not shown) produced no discernible phenotype compared to uninjected controls (Figure 3C and 3E). The specificity of xNor-MO was further tested by the co-injection of a wild-type *xNorrin* mRNA lacking the xNor-MO target sequence. The injection of 25 pg of *xNorrin* mRNA significantly rescued the anterior neural development defects in xNor-MO-injected embryos (Figure 3F) (*n* = 81, 77% rescued). Furthermore, the injection of xNor-MO into one dorsal animal cell in eight-cell-stage embryos, while leaving the other side intact, resulted in severe defects in eye development at later stages (compare Figure 3G and 3H). Because *xNorrin* is also expressed zygotically at later stages, we designed a splicing MO (spMO) to specifically block its splicing (Figure S3A and S3B). While xNor-MO inhibited anterior development, xNor-spMO had almost no effect on axis development (Figure S3C–S3G). We further confirmed that xNor-MO preferentially inhibited *XBF-1* (an anterior neural marker [26]) expression in the injected side, while xNor-spMO had a much weaker effect (Figure S3H). Neither MO had a significant role in regulating the expression of *HoxB9* (a posterior marker [27]) (Figure S3H). These results suggest that maternal *xNorrin* signaling is required for anterior CNS formation.

The loss of anterior head development may be an indirect effect due to a lack of early neuroectoderm specification. Thus, we tried to address whether β-catenin activation in the ectoderm, which is indispensable for full dorsal axis formation [3], depends on xNorrin activity. First, we used a SuperTopFlash Wnt reporter, which can be activated by injection into the dorsal animal blastomeres of eight-cell-stage embryos [5] (Figure 3I). The co-injection of xNor-MO with the reporter plasmid largely blocked reporter activity compared to co-injection with xNor-misMO (Figure 3I). In a separate assay, we examined whether maternal xNorrin was required for the expression of *Chordin*, *Noggin*, *Xnr3*, and *Siamois* in dorsal animal cells, which is one of the earliest indications of β-catenin activation [16]. We found that xNor-MO reduced the expression of these genes in late blastula embryos (Figures 3J, S4A and S4B) but did not interfere with the expression of *gsc* or *Xnr1* (Figure S4A and S4B) at the early gastrula stage. The reductions in the expression of these genes in xNor-MO embryos can be rescued by the co-expression of xNorrin (Figure S4B). In late blastula xNor-MO embryos (stage 9.5), the reduction of *Chordin* expression was mostly restricted to the ectoderm, while deep dorsal mesoderm cells retained weak expression (Figure 3L). The ectoderm expression of *Chordin* in the later blastula was fully restored by the co-injection of wild-type *xNorrin* mRNA lacking the MO target sequence (Figure 3N). In the blastula ectoderm, *Chordin* expression is controlled by maternal β-catenin [16],[28]. Thus, the control of early *Chordin* expression by xNorrin should partially reflect how xNorrin functions in neuroectoderm precursors. Together, these results indicate that, besides vegetally localized Wnt11 activity, maternal xNorrin is required to activate the canonical Wnt pathway in the dorsal ectoderm and is essential for the proper expression of early zygotic neural inducers before gastrulation.

### xNorrin-Activated Wnt Signaling Fails to Dorsalize Ventral Mesoderm

Mouse Norrin is a secreted protein that is tightly associated with the extracellular matrix [29]. However, we found that xNorrin was secreted into culture medium when expressed in HEK293 cells and *Xenopus* embryo explants (data not shown). The secretion of xNorrin in the culture cells and its potent activity in early embryos prompted us to speculate that other mechanisms may be required to restrict xNorrin activity in early embryos.

**Figure 4:**
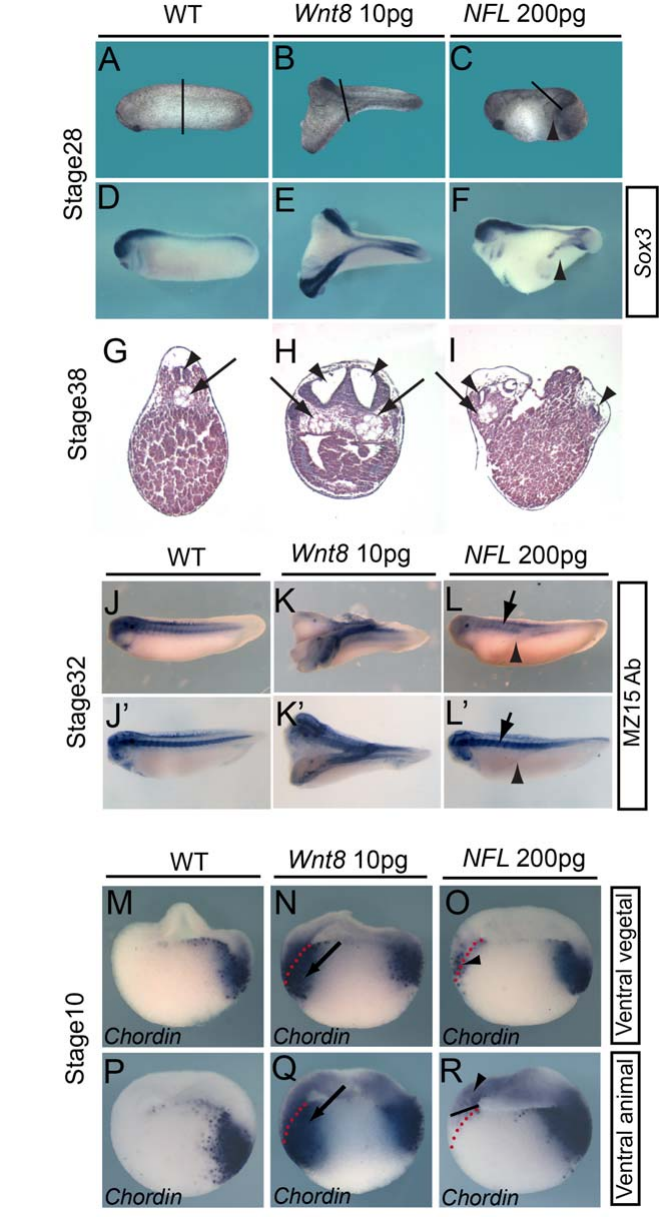
xNorrin activity is restricted to the ectoderm. (A–F) Injection of *NFL* into ventral vegetal cells at the eight-cell stage induced partial secondary axes. Wild-type (WT) embryos (stage 28) (A and D). Injection of *Wnt8* mRNA (10 pg) into ventral vegetal cells in eight-cell embryos induces complete secondary axis formation (stage 28) (69% of injected embryos showed secondary axes, *n* = 85) (B and E). *NFL* mRNA (600 pg total; 200 pg each) co-injection of ventral vegetal cells in eight-cell embryos induces partial secondary axis formation (stage 28) (45% of co-injected embryos showed secondary axes, *n* = 105) (C and F). Whole-mount in situ hybridization for *Sox3* in stage 28 embryos (D–F). Note that *NFL* induces *Sox3* expression in the partial secondary axis (arrowhead in [F]). The black lines in (A–C) indicate the section planes in (G–I), respectively. (G–I) Histological sections of embryos at stage 30. A wild-type embryo (G). *Wnt8* mRNA injection induced both secondary neural tube and notochord (H). *NFL* co-injection induced secondary neural tube but not notochord formation (I). Arrowhead: neural tube; arrow: notochord. (J–L) Immunostaining of notochords with the monoclonal antibody MZ15. Wild-type embryo with single notochord (all examined embryos) (J and J'). An embryo injected ventrally with *Wnt8* mRNA (10 pg) showed two notochords (induced secondary notochord and the primary notochord) (all examined embryos with secondary axes) (K and K'). An embryo injected ventrally with *NFL* (200 pg each) showed only the primary notochord (arrows), with notochord tissue absent in the partial secondary axis (arrowheads) (all examined embryos with partial secondary axes) (L and L'). Embryos are at stage 30. (M–R) *NFL* and *Wnt8* induced *Chordin* expression in different domains in the ventral cells. Whole-mount in situ hybridization was used to evaluate *Chordin* expression in stage 10 embryos. *Chordin* expression in wild-type early gastrula (M and P). *Wnt8* mRNA (10 pg) injection into ventral vegetal cells induced *Chordin* expression both in the superficial layer and in the deep layer of the marginal zone (87.5% of injected embryos showed the expression in both layers, *n* = 24) (N). Injection of *NFL* mRNAs (200 pg each) into the same domain induced *Chordin* expression mainly in the superficial layer (76% of injected embryos showed the expression, *n* = 30) (O). Injection of *Wnt8* into the ventral-animal cells of eight-cell embryos induced *Chordin* expression both in the ectoderm and in the mesoderm (84% of injected embryos showed the expression in both layers, *n* = 25) (Q). Injection of *NFL* mRNAs (200 pg each) into the same domain of eight-cell embryos induced *Chordin* expression only in the ectoderm (72% of injected embryos showed the expression, *n* = 32) (R). Arrowheads: superficial layer (O) or ventral ectoderm (R); arrows: deep layers on the ventral side. All embryos in (M–R) are shown with their dorsal sides to the right. Dotted lines delineate the superficial layer and deeper layer on the ventral side.

We first tested whether xNorrin was active when expressed ectopically in embryos. Previous studies indicated that the ectopic activation of the canonical Wnt pathway in the ventral side of early embryos is sufficient for secondary dorsal axis formation [30]–[32]. In addition, the co-expression of *NFL* was shown to activate canonical Wnt signaling in tissue culture cells and in animal cap explants (Figure S2B and S2C). Thus, we examined whether *NFL* could mimic canonical Wnt proteins and induce secondary axes in early embryos. Surprisingly, when injected into the ventral vegetal cells of early embryos, *NFL* failed to generate any complete secondary axes (Figure 4C), while *Wnt8* was able to generate secondary axes, as shown previously [31],[32] (Figure 4B). However, *NFL* was able to weakly induce partial secondary axes in which the neural marker *Sox3* was detected (Figure 4F). *NFL*-injected embryos had neural tubes but not notochords in their secondary axes (Figure 4I, 4L, and 4L'), while *Wnt8*-injected embryos had complete secondary axes containing both neural tubes and notochords (Figure 4E, H, K, and K') [31],[32].

The failure of *NFL*-injected embryos to form complete secondary axes was not due to a lack of activation of Wnt signaling by *NFL*, because *Chordin*, *Siamois*, and *Xnr3* expression could be detected in the ventral side of the early gastrula (stage 10) (Figure S4C and S4D). However, *Chordin* expression was mostly induced in the superficial layer and not in the deep ventral mesoderm (Figure 4O). The much lower expression of *Chordin* in the deep layer was considered unlikely to be a staining artifact because strong *Chordin* signal was readily detected in the dorsal mesoderm (Figure 4O). Embryos injected ventrally with *Wnt8* strongly induced *Chordin* expression in both germ layers, as expected (Figure 4N and 4Q). Similarly, the injection of *NFL* into ventral animal cells induced *Chordin* transcription only in the ventral ectoderm and not in the mesoderm (Figure 4R).

These results suggest that an intrinsic mechanism may exist to restrict endogenous xNorrin activity to the prospective neuroectoderm. Alternatively, injected *NFL* may alter the cell fate of endomesoderm, making it incompetent to form dorsal endomesoderm, even in the presence of canonical Wnt signaling.

### xNorrin Inhibits Activin/Nodal-Related Induced Mesoderm Formation

**Figure 5:**
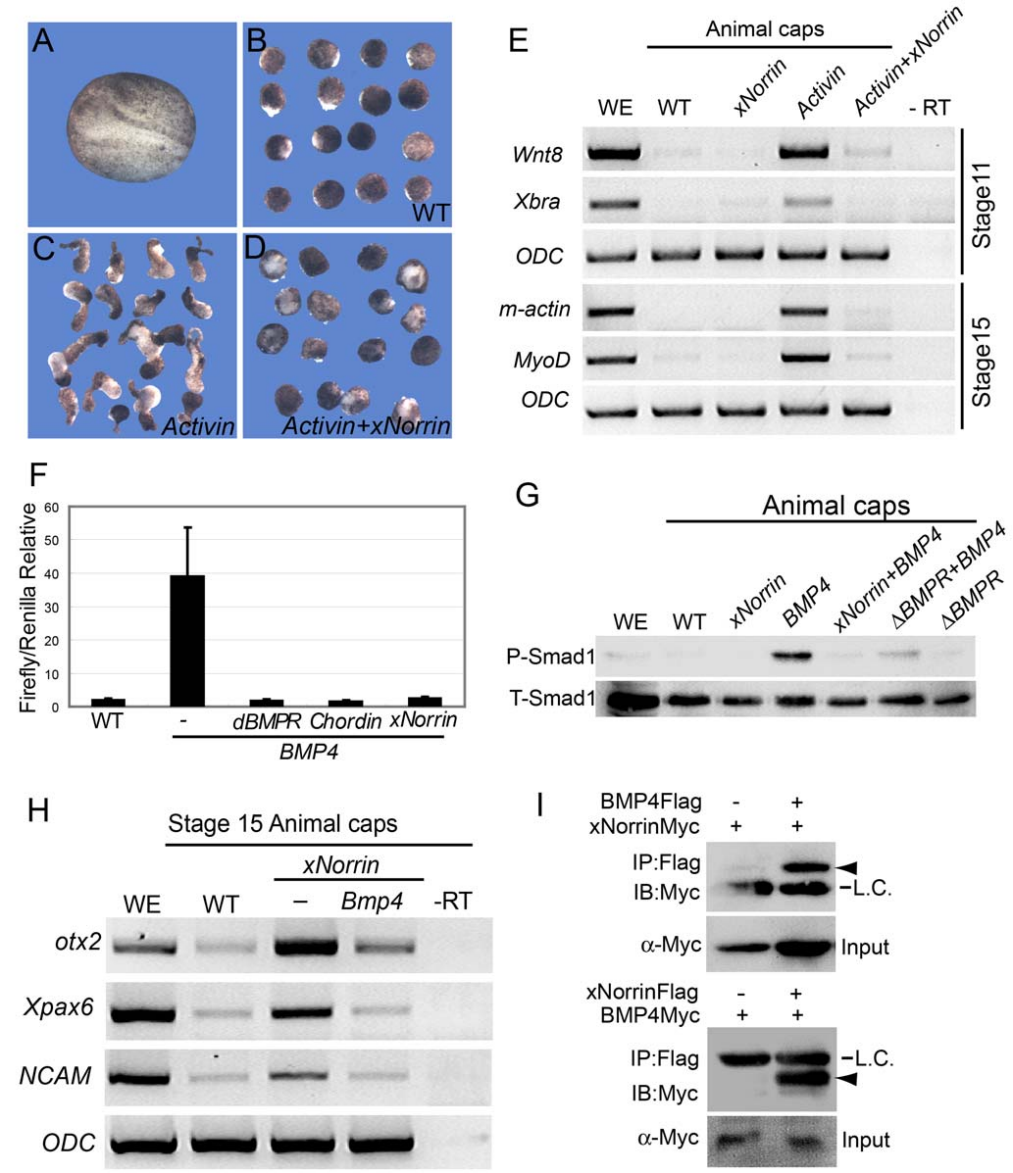
Reciprocal inhibition between xNorrin and TGF-β. (A–E) xNorrin inhibits *Activin-B*-mRNA-induced mesoderm formation. A wild-type (WT) embryo at a neurula stage (A). Wild-type animal caps with elongation (5% of the caps showed elongation, *n* = 60) (B). Elongated animal caps induced by *Activin-B* mRNA (25 pg) injection (82% of the injected caps showed elongation, *n* = 55) (C). Animal cap elongation was blocked in animal caps injected with *Activin-B* (25 pg) and *xNorrin* (200 pg) mRNAs (10% of the co-injected caps showed elongation, *n* = 58) (D). The *Activin-B*-mRNA-induced expression of mesoderm markers (*Wnt8*, *Xbra*, *m-actin*, and *MyoD*) was inhibited by xNorrin (E). RNAs were injected into the animal pole of one-cell embryos, and animal caps were cut around stage 8 and cultured in 1× MMR until the sibling embryos reached neurula stage. (F and G) xNorrin inhibits BMP4 signaling. *xNorrin* mRNA (500 pg), like Δ*BMPR* mRNA (200 pg) and *Chordin* mRNA (100 pg), inhibited BRE-Luc reporter activity in *Xenopus* embryos (F). *xNorrin* mRNA (500 pg) inhibited BMP4-induced Smad1 phosphorylation in animal caps (G). P-Smad1, phosphorylated Smad1; T-Smad1, total Smad1; WE, whole embryo. (H) BMP4 inhibited xNorrin-induced *otx2*, *Xpax6*, and *NCAM* RNA expression in animal caps of stage 15 embryos. –RT, no reverse transcription; WE, whole embryo; WT, wild-type animal caps. (I) xNorrin interacts with BMP4. *BMP4-Flag* and *xNorrin-Myc* mRNAs or *xNorrin-Flag* and *BMP4-Myc* mRNAs were injected into adjacent cells of four-cell embryos. FLAG-tagged proteins were immunoprecipitated (IP) from later gastrula embryos with a FLAG antibody. The proteins were PAGE separated and immunoblotted (IB) with an anti-c-Myc antibody. Arrowheads indicate xNorrin-Myc (top) or BMP4-Myc (bottom). L.C., IgG light chain.

Because *NFL* injection failed to activate Wnt target genes in the endomesoderm (Figure 4O and 4R), we initially proposed that an xNorrin-specific inhibitor might exist in the endomesoderm. However, after extensive investigation, we were not able to identify any molecule that could fulfill the proposed criteria for the inhibitor, i.e., that it should be expressed specifically in the endomesoderm and exert its antagonizing activity on xNorrin but not Wnt8. We thus turned to an alternative possibility, that *NFL* may influence the fate of endomesoderm precursor cells, making the germ layer incapable of conversion into dorsal endomesoderm. Previously, TGF-β family members, such as Xnr1, -2, -4, -5, and -6 and derriere were shown to be essential for mesoderm induction in *Xenopus* embryos [33]. Zygotic transcription of *Xnr* genes is activated by maternal transcription factor VegT and β-catenin. The Nodal-related molecules form a dorsal–ventral gradient that induces dose-dependent endomesoderm formation. Higher concentration of Nodal-related molecules results in dorsal specification [34],[35]. In a mesoderm induction assay, we used Activin, in lieu of Nodal-related molecules, to induce strong axial mesoderm and convergent extension in animal cap cells (Figure 5A–5C) [33],[36]. When co-expressed in animal cap cells, xNorrin completely blocked the Activin-induced elongation of animal cap explants (Figure 5D; compare to Figure 5B and 5C). The inhibition of mesoderm formation was confirmed by the lack of expression of the mesoderm markers *Xbra*, *Xwnt8*, *MyoD*, and *m-actin* in the co-expressing explants (Figure 5E). In whole embryos, xNorrin injection into the vegetal pole also blocked *Xbra* expression (Figure S5A–S5D). These results suggest that xNorrin may negatively regulate mesoderm induction in vivo. Next, we tested whether Xnr1 and xNorrin could be directly associated extracellularly. We combined and incubated conditioned medium from *Xnr1*-transfected HEK293 cells and from *xNorrin*-transfected cells and used the medium for immunoprecipitation. Indeed, we detected an association between Xnr1 and xNorrin (Figure S6B), suggesting that maternal xNorrin may restrict Nodal-related activity from extending into the animal pole.

### Reciprocal Inhibition between xNorrin and BMP4

Because xNorrin can inhibit Activin/Nodal-related activity, we hypothesized that it may also antagonize other members of the TGF-β superfamily. Indeed, we found that xNorrin also strongly inhibited the activity of a BMP4 reporter (BRE-Luc) (Figure 5F). As expected, xNorrin also inhibited Smad1 phosphorylation induced by BMP4 (Figure 5G). One possible mechanism for inhibition between proteins is through direct binding. We examined this possibility between BMP4 and xNorrin. To this end, we injected differently tagged *BMP4* and *xNorrin* mRNAs into adjacent blastomeres in advanced four-cell-stage embryos to allow secretion of the respective proteins into the extracellular space. At late gastrula, protein extract was immunoprecipitated with one tag antibody and blotted with the other tag antibody. Results showed that BMP4 was indeed associated with xNorrin extracellularly. Thus, the inhibition by xNorrin is likely through direct binding to BMP4 (Figure 5I).

The direct interactions between xNorrin and BMP4 led us to investigate whether xNorrin activity was regulated by BMP4. We showed that xNorrin induced neural marker expression in animal caps (Figure 5H). In an animal cap assay, BMP4 significantly inhibited the *otx2*, *Xpax6*, and *NCAM* expression induced by xNorrin (Figure 5H). Thus, reciprocal inhibition between xNorrin and BMP4 may also be implicated in dorsal–ventral ectoderm development.

**Figure 6:**
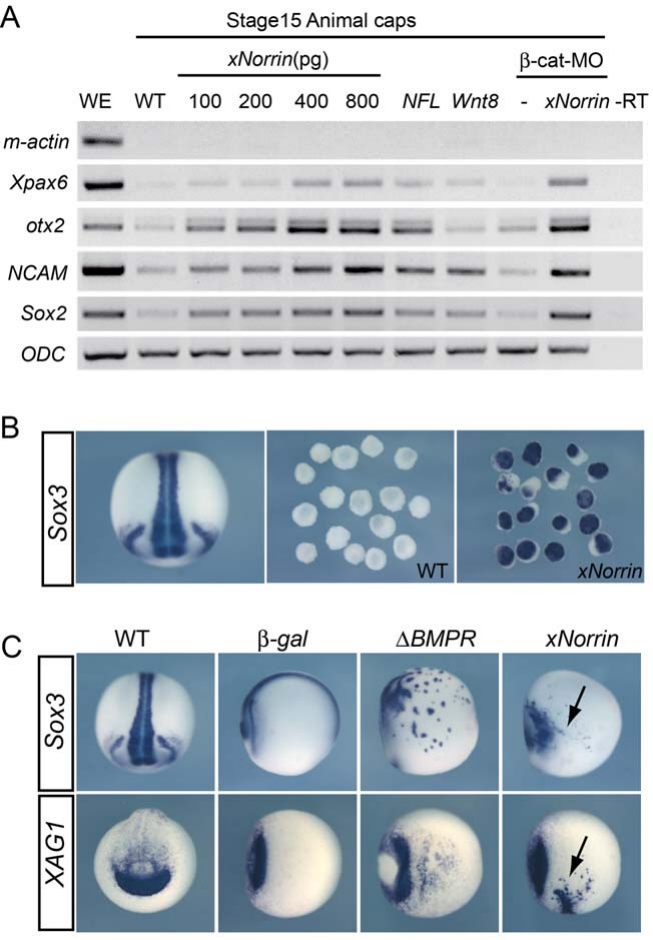
xNorrin induces neural formation independent of β-catenin signaling. (A) *xNorrin* dose-dependently induced neural marker (*Xpax6*, *otx2*, *NCAM*, and *Sox2*) expression. The induction is independent of mesoderm formation (*m-actin*: muscle actin) and could occur in the presence of a β-catenin-MO. RNA and MO were injected at the one-cell stage, and the caps were dissected around stage 8 and cultured until they reached stage 15. –RT, no reverse transcription; WE, whole embryo; WT, wild-type. (B) xNorrin induced *Sox3* expression in animal caps (89% of *xNorrin* injected caps showed the expression, *n* = 45). *xNorrin* mRNA (300 pg) was injected into the animal pole of two-cell embryos. *Sox3* expression in stage 15 animal caps was analyzed with in situ hybridization. WT, wild-type animal caps; xNorrin, *xNorrin*-mRNA-injected caps. (C) xNorrin induced the expression of ectopic neural and anterior markers in whole embryos. *xNorrin* mRNA (300 pg) was injected into the ventral animal tier cells of 32-cell embryos. Expression of *Sox3* (by Δ*BMPR*: 80%, *n* = 30; by *xNorrin*: 73%, *n* = 30) and *XAG1* (by Δ*BMPR*: 69%, *n* = 35; by *xNorrin*: 79%, *n* = 34) were induced at ectopic sites. β-gal, *β-gal*-mRNA-injected embryos; ΔBMPR: Δ*BMPR*-mRNA-injected embryos; WT, wild-type uninjected embryos; xNorrin: *xNorrin*-mRNA-injected embryos. Arrows: ectopically induced *Sox3* or *XAG1*. *Sox3* expression in wild type is shown in a dorsal view, while *XAG1* expression in wild type is shown in an anterior view. All other embryos are shown in a ventral view, except the embryo in the *β-gal*/*Sox3* panel, which is in a lateral view.

Previous studies indicated that the dorsally expressed BMP4 inhibitors Chordin, Noggin, and Follistatin could induce neural formation through direct binding [10]–[12]. Because xNorrin can also inhibit BMP4, we investigated xNorrin neural induction activity. Indeed, we found that xNorrin alone can induce the expression of neural-specific genes in animal cap cells in a dose-dependent manner (Figures 6A and S5A). The neural promoting activity of xNorrin in ectodermal cells was confirmed by the *Sox3* (a neural marker) expression in *xNorrin*-injected animal caps (Figure 6B). Further, xNorrin, like the truncated BMP receptor ΔBMPR, can induce ectopic *Sox3* and *XAG1* (an anterior marker) expression when injected into one ventral blastomere of 32-cell embryos (Figure 6C). More importantly, neural induction was observed when a β-catenin-specific MO was co-injected, indicating that canonical Wnt pathway activation is not required for neural formation in this setting (Figure 6A, compare the two β-catenin-MO-injected lanes). Furthermore, we did not observe activation of *Xnr3* or *Siamois* in *xNorrin*-injected animal caps, confirming the lack of canonical Wnt activation (Figure S2B). We conclude that xNorrin and BMP4 are reciprocally inhibited and that xNorrin may promote neural development independent of Wnt signaling activation.

### Loss of TGF-β Inhibition in a Subset of Norrin Mutants

Norrin mutations are responsible for both X-linked familial exudative vitreoretinopathy (FEVR) and Norrie disease (Online Mendelian Inheritance of Man MIM#310620) in humans [37],[38]. The finding that Norrin can inhibit BMP/TGF-β activity prompted us to test whether this regulation is involved in the disease development. We noticed that some previously identified Norrin mutants isolated from human patients did not significantly affect Wnt pathway activation [18],[39]. We hypothesized that these human Norrin mutants might instead be compromised in their ability to antagonize BMP/TGF-β activities in vivo.

**Figure 7:**
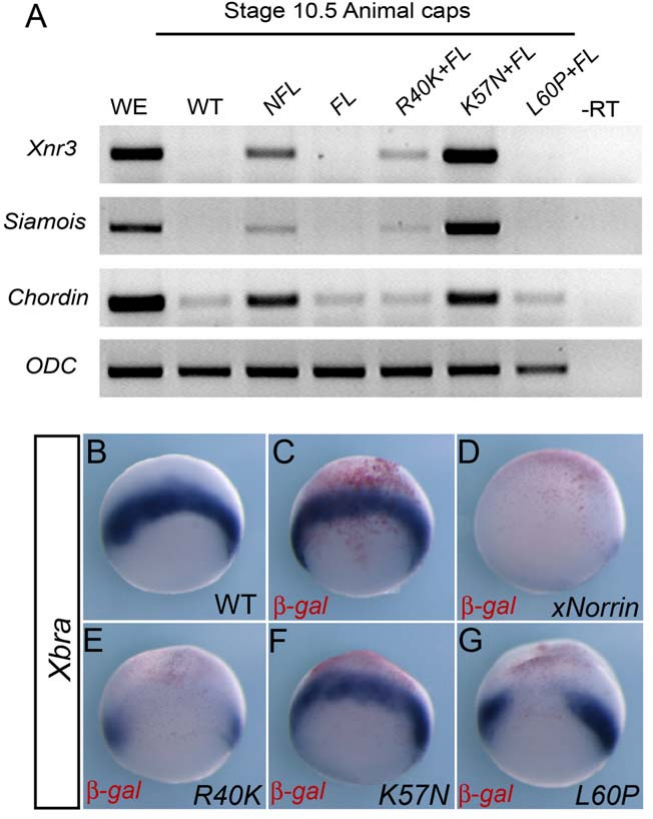
TGF-β inhibition is implicated in Norrie disease. (A) xNorrin point mutants showed various levels of Wnt activation activity. Wild-type (WT) or mutant *xNorrin* and *Fizzled4* and *Lrp5* (FL) were injected into animal poles. The expression of *Xnr3*, *Saimois*, and *Chordin* in animal caps was analyzed by RT-PCR. xNorrin R40K and xNorrin L60P showed slightly decreased and no Wnt activation, respectively. xNorrin K57N moderately increased Wnt activation. –RT, no reverse transcription; WE, whole embryo. (B–G) xNorrin point mutants showed various levels of mesoderm inhibition activity. Individual *xNorrin* point mutant mRNAs and *β-gal* mRNA were co-injected into the vegetal halves of two-cell-stage embryos. The expression of *Xbra* was analyzed at stage 10.5 by whole-mount in situ hybridization. While wild-type xNorrin inhibited *Xbra* expression (83% of the injected embryos showed very low or no *Xbra* expression, *n* = 35) (D), xNorrin K57N failed to inhibit *Xbra* expression (13% of the injected embryos showed reduced *Xbra* expression, *n* = 39) (F). xNorrin R40K (61% of the injected embryos, *n* = 33) and L60P (41% of the injected embryos, *n* = 32) also showed decreased *Xbra* expression (E and G). Uninjected (B) and *β-gal*-injected embryos (C). β-gal is stained in red. The embryos are in vegetal views, but slightly tilted toward marginal zones to show *Xbra* signal.

The ectopic expression of *xNorrin* or mouse *Norrin* in the vegetal cells of whole embryos potently inhibited the expression of the mesoderm-specific marker *Xbra*, which is dependent on Nodal-related activity in vivo (Figure 7D compared to 7B and 7C; Figure S5B–S5D). We thus used this assay to examine the activity of various xNorrin mutants on *Xbra* expression. We constructed three xNorrin point mutants (R40K, L60P, and K57N) based on mutations identified from human patients. Compared to wild-type xNorrin, the xNorrin R40K and L60P mutants showed decreased *Xnr3*, *Siamois*, and *Chordin* expression when co-expressed with Lrp5 and Frizzled4 in animal caps, while xNorrin K57N strongly activated these Wnt target genes (Figure 7A). This is consistent with previous findings using cell culture assays [18],[39]. In a whole-embryo assay, the xNorrin R40K mutant largely inhibited *Xbra* expression, while the xNorrin L60P mutant showed only slight inhibitory activity (Figure 7E and 7G compared to Figure 7B and 7D). In an extreme case, the xNorrin K57N mutant completely failed to suppress *Xbra* expression (Figure 7F). A lack of BMP4 binding ability might explain this loss of TGF-β inhibition. However, only a minor reduction in BMP4 binding was observed for the xNorrin K57N mutant compared to wild-type xNorrin. The xNorrin R40K mutant also did not show significantly reduced binding to BMP4 (Figure S6).

Next, we examined whether a lack of TGF-β inhibition by xNorrin compromised its neural induction function in a loss-of-function background. Because we could not directly study the K57N mutation through a knock-in experiment in *Xenopus*, we tested K57N mutant function in xNor-MO-injected embryos. In contrast to wild-type *xNorrin*, which was able to significantly rescue the anterior defects of the morphants (including eyes in 23% of the embryos) (Figure S7A–S7D), the K57N mutant was far less efficient, often producing phenotypes similar to those of xNor-MO-injected embryos (Figure S7E). We did not observe normal eye formation in any K57N-mutant-injected embryos (Figure S7F), suggesting that TGF-β inhibition is crucial for the full activity of xNorrin.

Together, these results indicate that Wnt activation and TGF-β inhibition activities are encoded by distinct domains in Norrin proteins and that the loss of TGF-β inhibition in Norrin mutants may be a novel mechanism implicated in the development of Norrie disease in humans (see Discussion).

## Discussion

**Figure 8:**
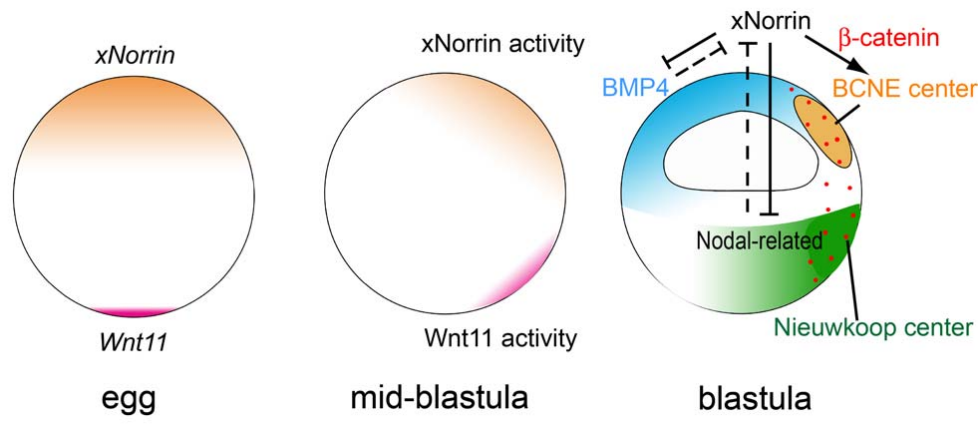
A model of dorsal specification in *Xenopus*. During oogenesis, maternal *xNorrin* and *Wnt11* are localized to the animal and vegetal poles, respectively. After fertilization, both mRNAs are enriched at the dorsal side, leading to two localized activity domains: BCNE center and Nieuwkoop center. xNorrin in the dorsal animal cells helps to specify neuroectoderm fate by activating a Wnt/β-catenin signaling domain, the BCNE center, and also participates in antagonizing the Nodal-related signal from the vegetal half and the BMP signal from the ventral side. Wnt11 in the dorsal vegetal domain is required for β-catenin activation in all dorsal regions, including in the BCNE center. Yellow: xNorrin and BCNE center. Purple: Wnt11. Red dots indicate stabilized β-catenin. Green: Nieuwkoop center.

The present work addresses the molecular nature and mechanism of a maternal signal that specifies the early neuroectoderm. Our findings reveal an essential coordination of canonical Wnt signaling activation and extracellular BMP/TGF-β inhibition by maternal xNorrin and further highlight the integration of the two major signaling pathways during early neuroectoderm specification (Figure 8). Our results also point to the de-repression of BMP/TGF-β as a new molecular mechanism in Norrie disease.

### Wnt Signaling Induction by xNorrin and Early Neuroectoderm Specification

Canonical Wnt signaling activation in early embryos is essential for the initial dorsal specification [2],[25]. Heasman and colleagues previously provided strong evidence that Wnt11 and Wnt5A are endogenous ligands required for β-catenin signaling in all dorsal cells, including dorsal animal cells [5]–[7]. These important findings seem to indicate that any additional Wnt agonists specifically required for β-catenin activation in dorsal animal cells would be redundant. However, previous studies suggested that in *Xenopus*, an animal-to-vegetal signal was implicated in promoting neural fate before gastrulation, and dorsal animal cells from the blastula are able to develop into neural tissues cell-autonomously in culture [18],[28],[40]. Noggin and Chordin were discovered to act as neural inducers prior to gastrulation. We demonstrated a lack of β-catenin activation in xNor-MO-injected embryos, which strongly indicated that Wnt11 activity was not sufficient to compensate for the loss of xNorrin activity in vivo (Figure 3I). The severe neural tissue formation defect in xNor-MO embryos is likely due to a failure in the specification of the early neuroectoderm. The significant down-regulation of the dorsal marker *Chordin* supports this hypothesis (Figure 3J–3N).

If both Wnt11 and xNorrin are involved in dorsal specification, then why does maternal xNorrin, which is likely retained in Wnt11-depleted embryos, fail to compensate for the loss of *Wnt11* RNA in generating anterior dorsal formation [5]? It is possible that additional molecules are required for xNorrin function in dorsal animal cells. For example, cortical rotation may play a role in the activation of xNorrin signaling. In fact, we found that the dorsal enrichment of xNorrin was lost in UV-irradiated embryos (Figure 2B and 2C and data not shown). One possibility is that a vegetal signal, such as Wnt11, may be required to fully activate xNorrin signaling in the dorsal ectoderm during cortical rotation. Candidate targets of this vegetal signal may include *Xenopus* Frizzled4 and *Xenopus* LRP5, two known receptors for xNorrin [18]. Similarly, the absence of *Xnr3* and *Siamois* expression in *xNorrin*-injected animal caps can be attributed to the lack of functional xNorrin receptors, which are required for xNorrin signaling (Figure S2B).

### Reciprocal Inhibition between xNorrin and TGF-β

In early embryos, balanced signaling activities from opposite domains are critical for patterning the dorsal–ventral, anterior–posterior, and animal–vegetal axes. For example, in the *Xenopus* gastrula, ventral BMP molecules antagonize Chordin and Noggin from dorsal cells through direct binding in the extracellular space [1]. Similarly, mesoderm-promoting Nodal activity in the vegetal pole is negatively regulated by maternal TGF-β signaling inhibitors, such as Coco and Ectodermin, from the animal half [41],[42]. In addition, the competence of blastomeres to form neural and retinal progeny is repressed by endomesoderm-promoting factors in the vegetal pole [43].

Previously, Coco expressed at the animal pole was proposed as a competence factor to block Nodal signaling and ensure the correct patterning of the ectoderm [41]. Our results indicate that xNorrin also directly inhibits BMP/TGF-β signaling, likely through direct extracellular binding without the activation of Wnt signaling (Figures 5 and 6). It is possible that this BMP antagonizing activity is required to predispose the dorsal ectoderm toward neural fates before zygotic BMP inhibitors are expressed. Both maternal Coco and xNorrin are expressed in overlapping domains in the animal pole of *Xenopus* oocytes [41]. It would be interesting to investigate how distinct TGF-β antagonists are coordinated to modulate multiple TGF-β signaling pathways in vivo. Although both Coco and xNorrin are TGF-β antagonists, there is a clear difference in that Coco also functions as a Wnt inhibitor by some unknown mechanism, while xNorrin is a Wnt agonist.

We also showed that the ectopic expression of xNorrin in the vegetal-marginal regions inhibited mesoderm formation and blocked gastrulation (Figure 7D and data not shown), underscoring the importance of restricting xNorrin activity to the dorsal animal pole. This result seems to suggest that the xNorrin-mediated inhibition of mesoderm formation may account for the unexpected failure of *NFL* to induce complete secondary axes on the ventral side (Figure 4). However, we observed that the combination of the *xNorrin* K57N mutant, *Frizzled4*, and *Lrp5* also failed to induce secondary axes (data not shown), suggesting that an alternative mechanism must prevent *NFL* from inducing secondary axes (Figure 4) [18]. A putative Norrin-specific inhibitor in the endomesoderm other than TGF-β cannot be excluded.

The BMP/TGF-β inhibition function of xNorrin may be attributed to a predicted cysteine-knot domain in the carboxyl terminal (Figure S1) [44]. A previous bioinformatics study classified the putative Norrin cysteine-knot domain as a mucin protein, along with secretory mucin and von Willebrand factor [45]. Other members of this subgroup may be tested for their potential ability to negatively regulate TGF-β family members.

Conversely, BMP4 was shown to repress Norrin-induced neural formation (Figure 5H). In addition to *xNorrin* RNA localization in the dorsal animal region, ventrally expressed BMP4 and vegetally expressed mesoderm inducers, such as Nodal, may further restrict xNorrin activity to the prospective neuroectoderm. Thus, reciprocal inhibition between BMP/TGF-β and xNorrin are equally important for appropriate embryonic patterning.

### A Link between TGF-β Signaling and Norrie Disease

Norrin has been identified as an activator of the canonical Wnt signaling pathway through two separate receptor complexes, Frizzled4/Lrp5 and Frizzled4/TSPAN12 [17]–[19]. Given the direct link between Norrin mutations and Norrie disease, and the roles of TGF-β signaling in multiple human diseases, it is important to recognize that Norrin also functions as a potent inhibitor of TGF-β family members. Two lines of evidence indicate that canonical Wnt signaling and TGF-β inhibition are induced separately by Norrins. First, xNorrin can induce neural formation in the absence of Wnt target activation (Figure 6A). Second, selected xNorrin point mutants (e.g., K57N) potently suppress endogenous TGF-β target gene expression but maintain robust Wnt activation capability (Figure 7).

One of the major defects caused by Norrie disease is abnormal vascular development in the retina and inner ear [18]. The development of the elaborate vascular structure in the retina is strongly influenced by VEGF, which in turn can be positively regulated by TGF-β/ALK5 signaling [46]. Although Norrin mutations cause retinal hypovascularization, a previous study showed that the numbers of blood vessels in the ganglion cell layer and the nerve fiber layer are actually increased in the Norrin knock-out mice [47], suggesting a pro-angiogenesis activity that may be enhanced in these cell layers. Because Norrin hemizygous mutant mice also have severe defects in ear and brain development [18],[48], further investigation of local TGF-β regulation in these organs is also warranted.

Finally, the finding that two independent activities are encoded in the small Norrin protein (mature human Norrin has only 109 amino acid residues) raises the question of how Wnt pathway activation and TGF-β signal tuning are coordinated by Norrin in vivo. Solving the three-dimensional structures of the wild-type Norrin protein and selected point mutants may help answer this question and may even help elucidate the molecular mechanisms of Wnt agonist signaling through its receptors.

## Materials and Methods

### Plasmid and mRNA

The initial *xNorrin* cDNA clone was first amplified from a *X. laevis* cDNA library using Pyrobest DNA polymerase (TaKaRa) and PCR primers partially based on a predicted *X. tropicalis Norrin* gene sequence. (http://genome.jgi-psf.org/cgi-bin/dispGeneModel?db=Xentr4&id=158316): *xNorrin* Up: 5′-AGACGAATTCACCTGAGAGGAAGACTGGG-3′, *xNorrin* Down: 5′-AGACCTCGAGAGCAACGCAAGCGAATGG-3′. The cDNA for the coding region was amplified using *xNorrin* Up: 5′-AATCGGATCCATGGGAAATCGTGTCCTTC-3′ and *xNorrin* Down: 5′-ATATCTCGAGCTATGAATTGCACTCTTC-3′. The *xNorrin* cDNA was then cloned between the BamHI and XhoI sites of pCS2^+^. The *xNorrin5′-Myc* plasmid was generated by inserting *xNorrin* cDNA, including the 5′ UTR, between the BamHI and ClaI sites of pCS2^+^MT, thereby introducing a C-terminal Myc-tag. The pCS2^+^ *xNorrin*-FLAG plasmid was generated by inserting *xNorrin* cDNA between the BamHI and XbaI sites of pCS2^+^-FLAG-C4. The *xNorrin* single point mutants (R40K, K57N, and L60P), which mimic human Norrie disease mutants, were generated by site-direct mutagenesis (Fast Mutagenesis System, TransGen) [18].

The mRNA for *Xenopus* injections was prepared using the RiboMax Large Scale RNA Production System (Ambion) according to the manufacturer's instructions. The pCS2^+^-*xNorrin*, pCS2^+^-*xNorrin* (R40K, K57N, or L60P), *xNorrin-Myc*, pCS2^+^-*xFrizzled4* [49], pCS2^+^-*hLrp5* [50], and *Wnt8* [31] plasmids were all linearized with NotI; *BMP4* was linearized with EcoRI; and *Wnt11* [51] was linearized with BamHI. All plasmids were transcribed with SP6 RNA polymerase. RNA microinjections were carried out as described [52].

### Embryo Manipulation and Injection

All animal studies in this report were approved by the Institutional Review Board of the Institute of Genetics and Developmental Biology, Chinese Academy of Sciences. *X. laevis* eggs were isolated in 1× MBS plus high salt solution and fertilized using sperm suspensions in 1× MMR. Embryos were cultured in 0.1× MMR. Embryo dissection was performed as previously described [52]. Briefly, mid-blastula embryos were transferred into 1× Steinberg's solution, the vitelline membrane was removed, and 3×3 mm^2^ animal caps were cut. Explants were cultured in 1× Steinberg's solution until they reached the indicated stages [53].

For MO oligonucleotides and mRNA injections, embryos were transferred into 1× MMR containing 2% Ficoll (Amersham Biosciences). Pigment intensity was used to differentiate the dorsal and ventral sides. After injections, embryos were washed thoroughly and returned to 0.1× MMR during the blastula stage.

For UV treatment, embryos were irradiated by placing them in a quartz colorimetric cup oriented with the animal pole upwards and UV-irradiated at 50 µJ using the Stratagene Crosslinker 1800. Immediately after irradiation, the embryos were transferred into 1× MMR containing 2% Ficoll. For rescue experiments, four- to eight-cell-stage embryos were injected with 500 pg of *xNorrin* mRNA or 500 pg of *Wnt11* mRNA [23].

### Antisense Morpholinos

xNorrin antisense MOs were purchased from Genetools. The MO sequences used were: xNor-MO: CTCAATCCCAGTCTTCCTTTCAGGT, xNor-misMO: CTGAATCCGAGTGTTCGTTTCACGT, and xNor-spMO: TTAAAGTGGACTGTACCTTGGCAGT. MOs were dissolved in sterile, filtered water at a concentration of 5 ng/nl and injected at the doses described in the text.

### Reverse Transcription PCR

Total RNA was prepared using the Proteinase K method and treated with 10 µg of yeast tRNA and RNase-free DNase (Promega) before cDNA synthesis [52]. cDNA was synthesized by reverse transcription, and the reactions were performed in a volume of 20 µl using 200 ng of random primer (Promega), 5× first-strand buffer, 0.01 M DTT, 40 U RNase inhibitor (TaKaRa), 1 mM each dNTP, and 200 U M-MLV RT (Invitrogen) at 37°C for 50 min. Reactions were then heat-inactivated at 70°C for 10 min and stored at −20°C. One-tenth of the mixture was used as a template for PCR. PCR was carried out in a volume of 25 µl containing 100 mM dNTPs, 0.2 µM each primer, and 1 U of rTaq DNA polymerase (TaKaRa). The PCR parameters and DNA primers are described in Table S1. PCR cycles were determined such that no amplification saturation was reached in semi-quantitative assays.

### Luciferase Assays

SuperTopFlash DNA (20 pg), containing eight copies of the TCF-binding site upstream of a minimal TK promoter and the luciferase open reading frame, and pRL-TK DNA (10 pg) (*Renilla* luciferase was used as an internal control) [5] were co-injected into two dorsal animal cells at the eight-cell stage of wild-type, xNor-MO (20 ng)–injected, or xNor-misMO (20 ng)–injected embryos Three replicate samples for each of the three embryo types were frozen at the late blastula stage, and luciferase assays were carried out using a Promega Luciferase Assay system.

### Western Blot

To test for xNor-MO activity, one-cell-stage embryos were injected with 5 ng, 10 ng, or 20 ng of xNor-MO at the marginal zone and then injected four times with a total of 1.5 ng of *xNorrin-Myc* mRNA into the marginal zone at the four-cell stage. A total of five blastula embryos were homogenized in 100 µl of ice-cold lysis buffer [54]. Protein lysates were spun for 15 min at high speed at 4°C. Protein detection by Western blot was performed using anti-c-Myc (9E10) primary (Santa Cruz Biotechnology) and HRP-conjugated secondary (Pierce) antibodies with Pierce Western blot detection reagents.

For co-immunoprecipitation assays, 500 pg of *xNorrin-Myc* and 500 pg of *BMP4* mRNA were injected into different cells of four-cell-stage embryos. The injected embryos were frozen at stage 10 in batches of five and lysed with 500 µl of ice-cold lysis buffer. The cleared lysate was mixed with anti-FLAG-M2 agarose beads and incubated overnight at 4°C. The beads were pelleted, washed four times with lysis buffer, mixed with SDS-PAGE sample buffer, and processed in a standard electrophoresis and Western blot protocol.

### Whole-Mount In Situ Hybridization

Whole-mount in situ hybridization was performed according to a standard protocol as described previously [55], with minor modifications for dissected embryos. For dissected embryos, whole pigmented embryos were fixed for 1 h in MEMFA, bisected along the dorsal–ventral axis with a scalpel blade, fixed for two additional hours in MEMFA, and washed and stored in 100% methanol. The embryos were hybridized at 65°C overnight. BM Purple was used as a substrate (Roche). Pigment was then bleached. The RNA probes were labeled with digoxigenin-UTP (Roche) with the appropriate RNA polymerase using linearized plasmids.

### Histological Analysis

For histological analysis, embryos were fixed overnight in Bouin's solution and then dehydrated and embedded in paraffin. Sections of 10-mm thickness were prepared and stained with hematoxylin and eosin as previously described [56].

## Supporting Information

Figure S1 **Norrins are highly conserved in vertebrates.** (A) An alignment of Norrin protein sequences from selected vertebrate species. Prefixes used for Norrins from different species: human (h), mouse (m), chicken (c), *X. laevis* (xl), *X. tropicalis* (xt), and zebrafish (z). Conserved cysteine residues are highlighted in red. (B) Percentages of identical amino acid residues between Norrin proteins from different species.
(TIF)

Figure S2 **Maternal xNorrin activates the canonical Wnt pathway.** (A) *xNorrin* expression during early *Xenopus* development detected by reverse transcription PCR (RT-PCR). *ODC* (ornithine decarboxylase) served as a loading control. RT–: no reverse transcription. Embryos were staged according to Nieuwkoop and Faber [53]. (B) Expression of *Siamois* and *Xnr3* (Wnt target genes) and *Xbra* (mesoderm marker) in isolated animal caps from embryos co-injected with *NFL* (200 pg each), *xNorrin* (200 pg), or *Wnt8* (10 pg) mRNAs. Nor, Norrin; WE, whole wild-type embryo; WT, wild type. *ODC* served as a loading control. (C) Norrin can lead to phosphorylation of its receptor, LRP6. LRP6 phosphorylation at three specific threonine (T) and serine (S) sites (T1479, S1490, and T1493) was analyzed in HEK293 cells transfected with *Lrp6/Axin/mFz4*, with or without mouse *Norrin* or *xNorrin*, using site-specific antibodies. Total LRP was detected using a general LRP antibody.
(TIF)

Figure S3 **Maternal xNorrin is required for anterior neural formation.** (A) The genomic sequence of the first exon and the first intron boundary of xNorrin. The first two presumptive nucleotides “gt” of the intron are labeled in red. The splicing site sequence targeted by xNor-spMO is indicated by a green line. See Materials and Methods for sequence information for all MOs. (B) RT-PCR to detect *xNorrin* mRNA expression in stage 15 embryos. xNor-spMO (20 ng) inhibited zygotic *xNorrin* transcription, while xNor-MO (20 ng) or xNor-misMO (20 ng) (a four-nucleotide mismatched MO compared to xNor-MO) did not. (C–F) Representative MO-injected tadpole at stage 34. xNo-spMO (20 ng) did not cause severe anterior defects, unlike xNor-MO. (G) Summary of (C–F). Uninjected: *n* = 30; MO: *n* = 24; misMO: *n* = 30; spMO: *n* = 40. (H) xNor-MO injection inhibited anterior neural formation. Whole-mount in situ hybridization was performed for *XBF-1* mRNA (anterior neural marker) and *HoxB9* mRNA (posterior neural marker) in stage 15 embryos. Dorsal animal cell injection of xNor-MO (10 ng) at the four- to eight-cell stage greatly reduced the expression of the anterior neural marker *XBF-1* (63%, *n* = 32), while injection of xNor-misMO (10 ng) or xNor-spMO (10 ng) (27%, *n* = 29) was far less effective to reduce the expression. Neither xNor-MO (*n* = 25) nor xNor-spMO (*n* = 25) injection affected *HoxB9* expression. MOs were co-injected with *β-gal* mRNA (100 pg). β-gal staining is shown in red. *XBF-1* staining embryos are shown in anterior view, and *HoxB9* staining embryos are shown in dorsal view, with the anterior pole at the top. misMO, xNor-misMO; MO, xNor-MO; spMO, xNor-spMO.
(TIF)

Figure S4 **xNorrin is essential for early dorsal-specific gene expression.** (A) The injection of xNor-MO reduced *Xnr3* (stage 9: 70% reduced, *n* = 17; stage 10: 70% reduced, *n* = 20) but not *gsc* (14% reduced, *n* = 22) expression, as assayed by whole-mount in situ hybridization. (B) RT-PCR analysis showed that xNor-MO injected into dorsal animal cells reduced the expression of early dorsal-specific genes in stage 9 embryos. This reduction could be rescued by the injection of *xNorrin* mRNA (50 pg) lacking the xNor-MO target sequence. Note that xNor-MO did not change *Xnr1* expression. *ODC* served as a loading control. (C) The overexpression of NFL in ventral vegetal blastomeres induced Wnt target gene expression. Upon injection into the ventral blastomeres of early eight-cell embryos, both *NFL* and *Wnt8* induced *Xnr3* (*NFL*: 56%, *n* = 25; *Wnt8*: 83%, *n* = 46) and *Chordin* (*NFL*: 72%, *n* = 29; *Wnt8*: 88%, *n* = 60) expression. However, *NFL* only weakly induced the Spemann organizer marker *gsc* (*NFL*: 7%, *n* = 28; *Wnt8*: 84%, *n* = 50). All embryos are in vegetal views. Arrowheads indicate the injection sites. (D) RT-PCR results (not quantitative) showed that *NFL* injection into ventral-vegetal cells ectopically activated Wnt target genes (*Chordin*, *Siamois*, and *Xnr3*) at the ventral side of the embryos. *ODC* served as a loading control.
(TIF)

Figure S5 **xNorrin induces neural formation and inhibits mesoderm formation.** (A) RT-PCR analysis of neural gene expression in stage 15 animal caps from embryos injected with *Wnt8* (20 pg), *Wnt11* (200 pg), *xNorrin* (*xNor*) (200 pg), and *NFL* (200 pg each). The expression of *otx2*, *Sox2*, and *NCAM* (all neural markers) was analyzed. *ODC* served as a loading control. (B–D) *Xbra* expression detected in whole-mount in situ hybridization. A wild-type embryo at stage 10.5 (B); reduced *Xbra* expression in stage 10.5 embryos injected with *xNorrin* RNA (200 pg) at the vegetal pole at the two-cell stage (82% reduced, *n* = 34) (C); reduced *Xbra* expression in stage 10.5 embryos injected with mouse *Norrin* RNA (200 pg) at the vegetal pole at the two-cell stage (66% reduced, *n* = 36) (D).
(TIF)

Figure S6 **xNorrin interacts with TGF-β family members.** (A) xNorrin binds to BMP4. Epitope-tagged wild-type xNorrin or xNorrin point mutants (R40K or K57N) and BMP4 were separately expressed in HEK293 cells. The conditioned medium from cells expressing individual xNorrin and BMP4 were mixed and incubated. The FLAG-tagged protein complexes were immunoprecipitated using an anti-FLAG antibody and separated in SDS-PAGE and blotted. An anti-c-Myc antibody was used to detect Myc-tagged BMP4. The expression of FLAG-tagged proteins was detected using an anti-FLAG antibody. xNorrin was shown to bind to BMP4. The R40K mutant retained this binding activity, while the K57N mutant showed slightly reduced BMP4 binding. Conditioned medium of the parent FLAG-plasmid-transfected cells was used as a control (ctrl). Arrowhead, BMP4-Myc; arrow, immunoglobulin light chains (LC). α-FLAG, anti-FLAG tag monoclonal antibody; α-Myc, anti-c-Myc-tag monoclonal antibody. (B) xNorrin binds to Xnr1 but not DKK-1. The assay performed was similar to that described in (A). Conditioned medium (ctrl) was the same as in (A). Arrowheads indicate FLAG-tagged protein. Arrows point to immunoglobulin heavy chain (HC, top) and light chain (LC, bottom).
(TIF)

Figure S7 **The xNorrin K57N mutant failed to efficiently rescue anterior defects in xNorrin morphants.** (A–E) An uninjected embryo (A). A xNor-MO (20 ng)–injected embryo (B). Note the lack of eye pigment. A xNor-misMO (20 ng)–injected embryo (C). A xNor-MO and wild-type *Norrin* RNA (50 pg) co-injected embryo (D). A xNor-MO and *xNorrin* K57N RNA (50 pg) co-injected embryo (E). (F) Summary of anterior defect frequency in (A–E). Uninjected: *n* = 40; MO: *n* = 24; xNor rescue: *n* = 20; K57N rescue: *n* = 19; misMO: *n* = 15. RNAs and MO were injected into the dorsal animal region at the four-cell stage. All embryos shown are around stage 36.
(TIF)

## Abbreviations

BCNE center: blastula *Chordin-* and *Noggin*-expressing center
CNS: central nervous system
misMO: mismatched morpholino
MO: morpholino
*NFL*: *Xenopus Norrin* plus *Xenopus Frizzled4* plus human *Lrp5*
RT-PCR: reverse transcription PCR
spMO: splicing morpholino
UV: ultraviolet, xNorrin, *Xenopus* Norrin
