# Supplementary figures and images for: Maternal xNorrin, a Canonical Wnt Signaling Agonist and TGF-β Antagonist, Controls Early Neuroectoderm Specification in Xenopus
Source: PLoS Biol. 2012 Mar 20;10(3):e1001286. doi: 10.1371/journal.pbio.1001286 (PMC3308935; doi:10.1371/journal.pbio.1001286)

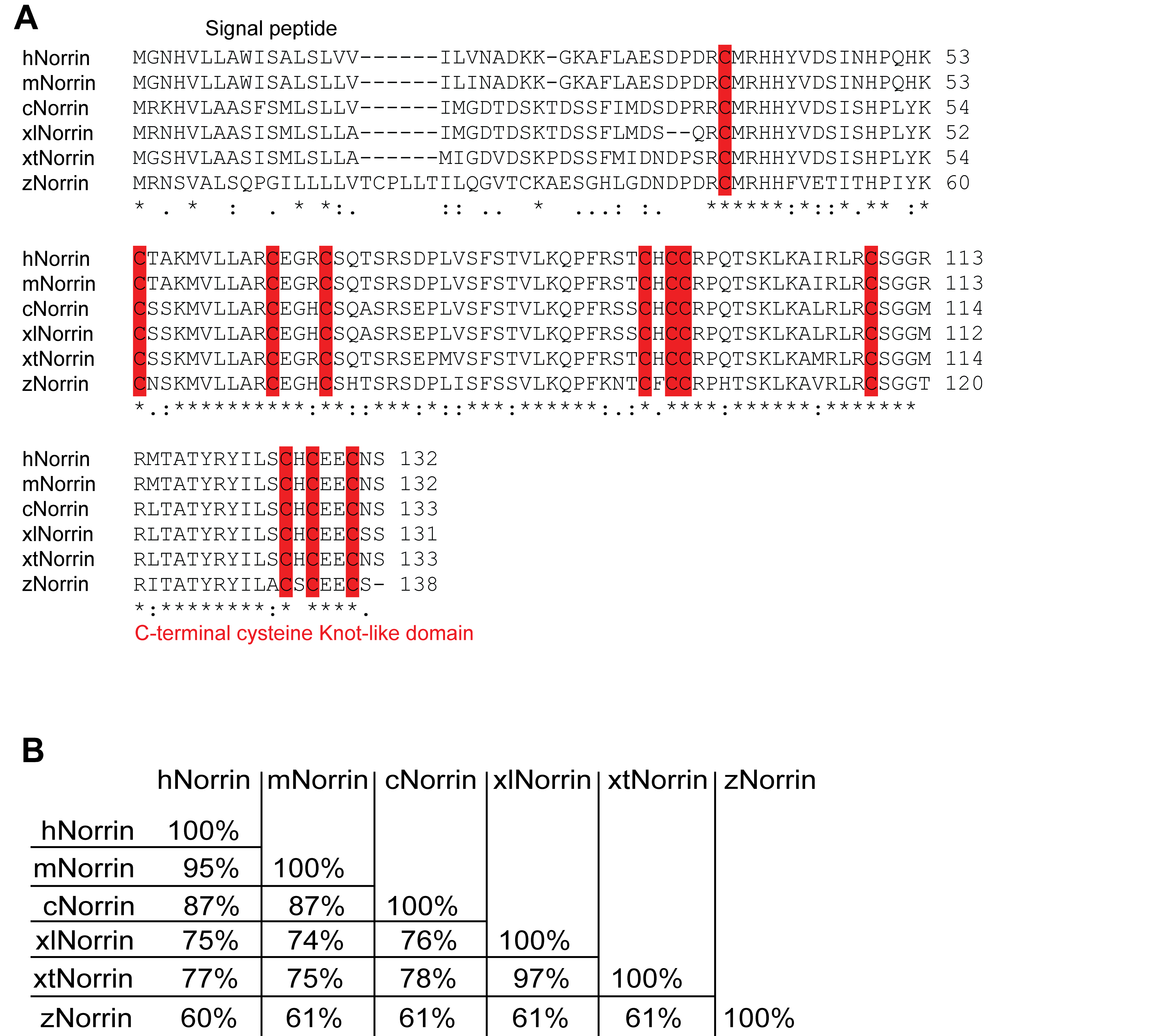

Supplement: Figure S1 — Norrins are highly conserved in vertebrates. (A) An alignment of Norrin protein sequences from selected vertebrate species. Prefixes used for Norrins from different species: human (h), mouse (m), chicken (c), X. laevis (xl), X. tropicalis (xt), and zebrafish (z). Conserved cysteine residues are highlighted in red. (B) Percentages of identical amino acid residues between Norrin proteins from different species. (TIF) [file pbio.1001286.s001.tif]

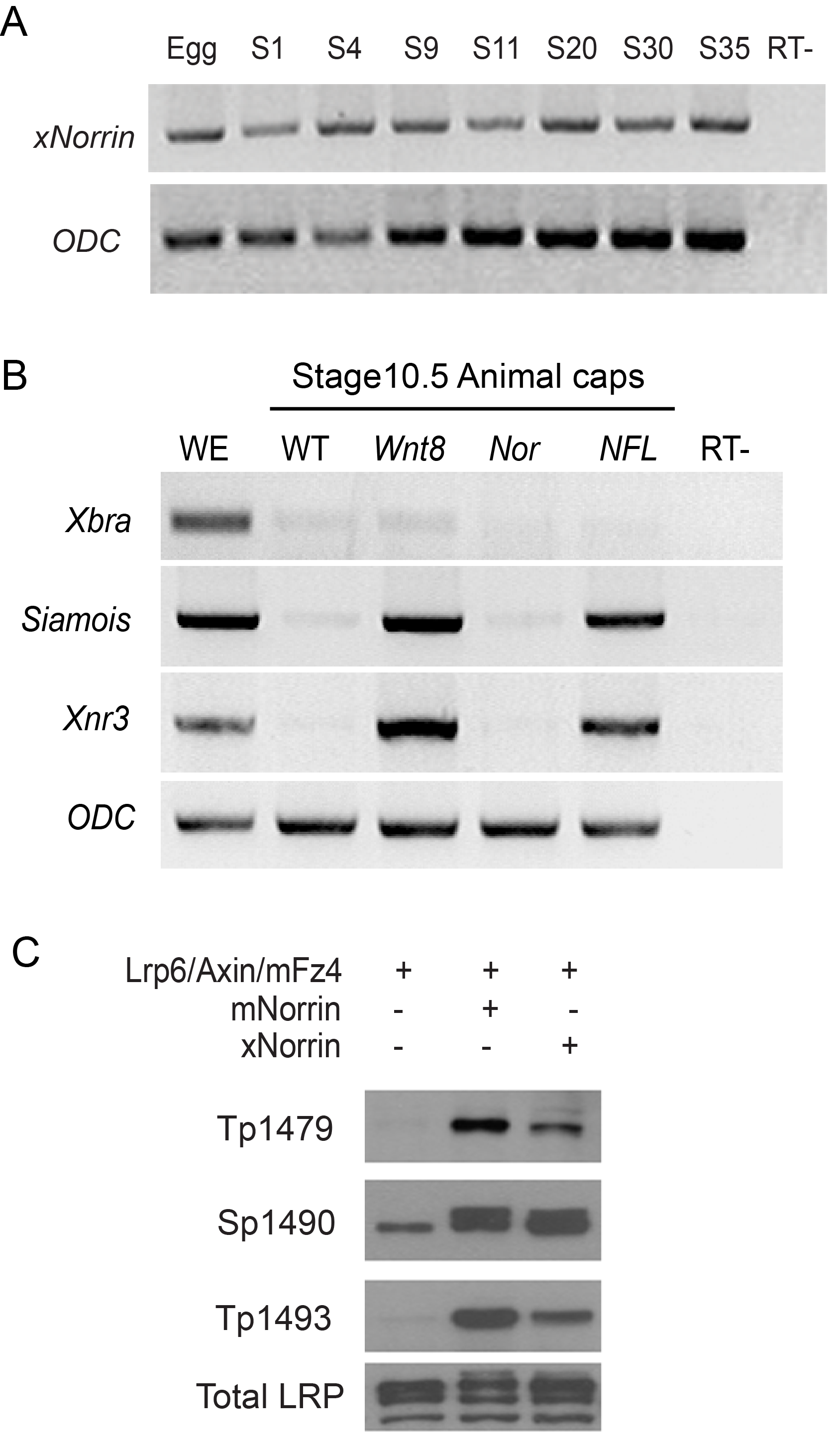

Supplement: Figure S2 — Maternal xNorrin activates the canonical Wnt pathway. (A) xNorrin expression during early Xenopus development detected by reverse transcription PCR (RT-PCR). ODC (ornithine decarboxylase) served as a loading control. RT–: no reverse transcription. Embryos were staged according to Nieuwkoop and Faber [53]. (B) Expression of Siamois and Xnr3 (Wnt target genes) and Xbra (mesoderm marker) in isolated animal caps from embryos co-injected with NFL (200 pg each), xNorrin (200 pg), or Wnt8 (10 pg) mRNAs. Nor, Norrin; WE, whole wild-type embryo; WT, wild type. ODC served as a loading control. (C) Norrin can lead to phosphorylation of its receptor, LRP6. LRP6 phosphorylation at three specific threonine (T) and serine (S) sites (T1479, S1490, and T1493) was analyzed in HEK293 cells transfected with Lrp6/Axin/mFz4, with or without mouse Norrin or xNorrin, using site-specific antibodies. Total LRP was detected using a general LRP antibody. (TIF) [file pbio.1001286.s002.tif]

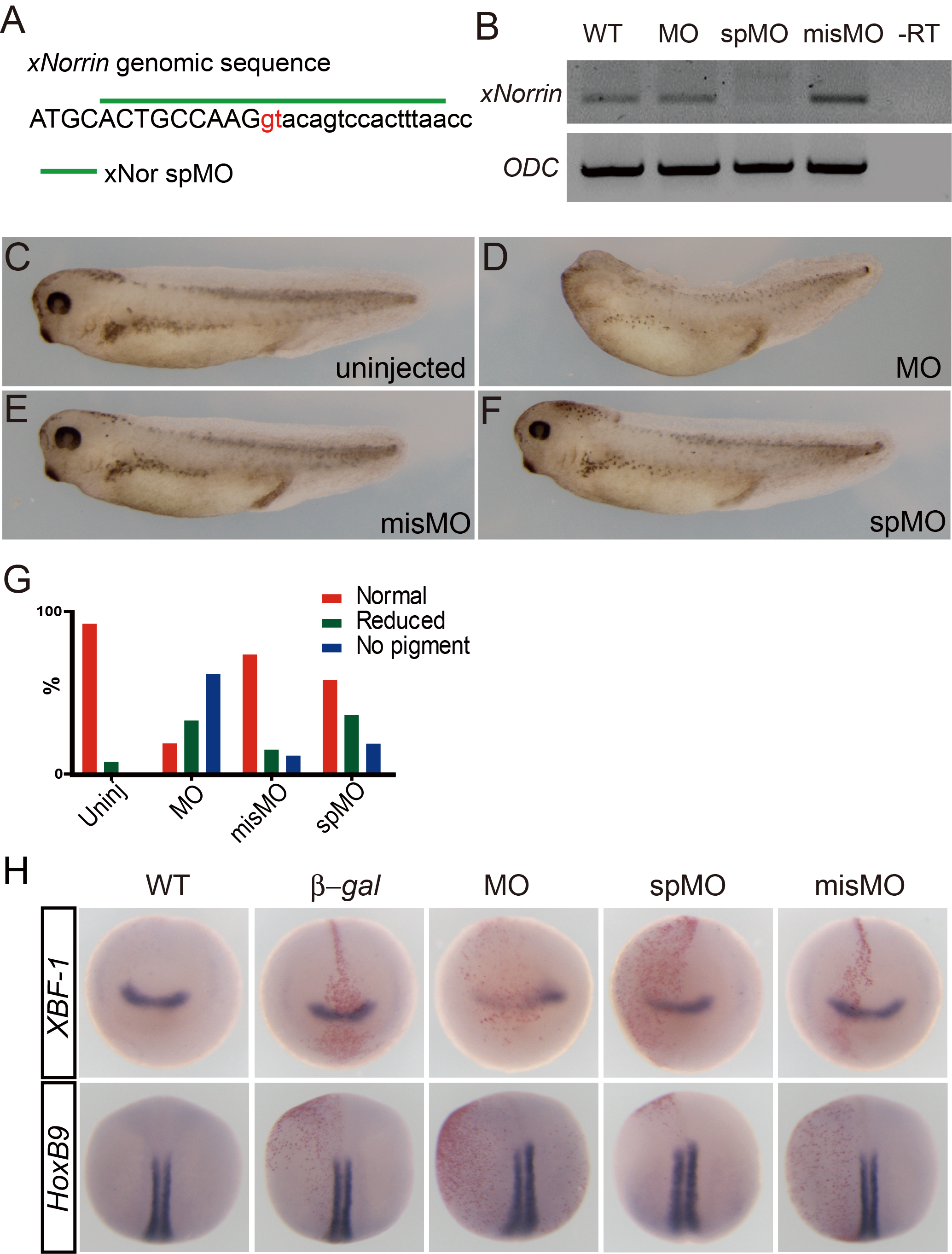

Supplement: Figure S3 — Maternal xNorrin is required for anterior neural formation. (A) The genomic sequence of the first exon and the first intron boundary of xNorrin. The first two presumptive nucleotides “gt” of the intron are labeled in red. The splicing site sequence targeted by xNor-spMO is indicated by a green line. See Materials and Methods for sequence information for all MOs. (B) RT-PCR to detect xNorrin mRNA expression in stage 15 embryos. xNor-spMO (20 ng) inhibited zygotic xNorrin transcription, while xNor-MO (20 ng) or xNor-misMO (20 ng) (a four-nucleotide mismatched MO compared to xNor-MO) did not. (C–F) Representative MO-injected tadpole at stage 34. xNo-spMO (20 ng) did not cause severe anterior defects, unlike xNor-MO. (G) Summary of (C–F). Uninjected: n = 30; MO: n = 24; misMO: n = 30; spMO: n = 40. (H) xNor-MO injection inhibited anterior neural formation. Whole-mount in situ hybridization was performed for XBF-1 mRNA (anterior neural marker) and HoxB9 mRNA (posterior neural marker) in stage 15 embryos. Dorsal animal cell injection of xNor-MO (10 ng) at the four- to eight-cell stage greatly reduced the expression of the anterior neural marker XBF-1 (63%, n = 32), while injection of xNor-misMO (10 ng) or xNor-spMO (10 ng) (27%, n = 29) was far less effective to reduce the expression. Neither xNor-MO (n = 25) nor xNor-spMO (n = 25) injection affected HoxB9 expression. MOs were co-injected with β-gal mRNA (100 pg). β-gal staining is shown in red. XBF-1 staining embryos are shown in anterior view, and HoxB9 staining embryos are shown in dorsal view, with the anterior pole at the top. misMO, xNor-misMO; MO, xNor-MO; spMO, xNor-spMO. (TIF) [file pbio.1001286.s003.tif]

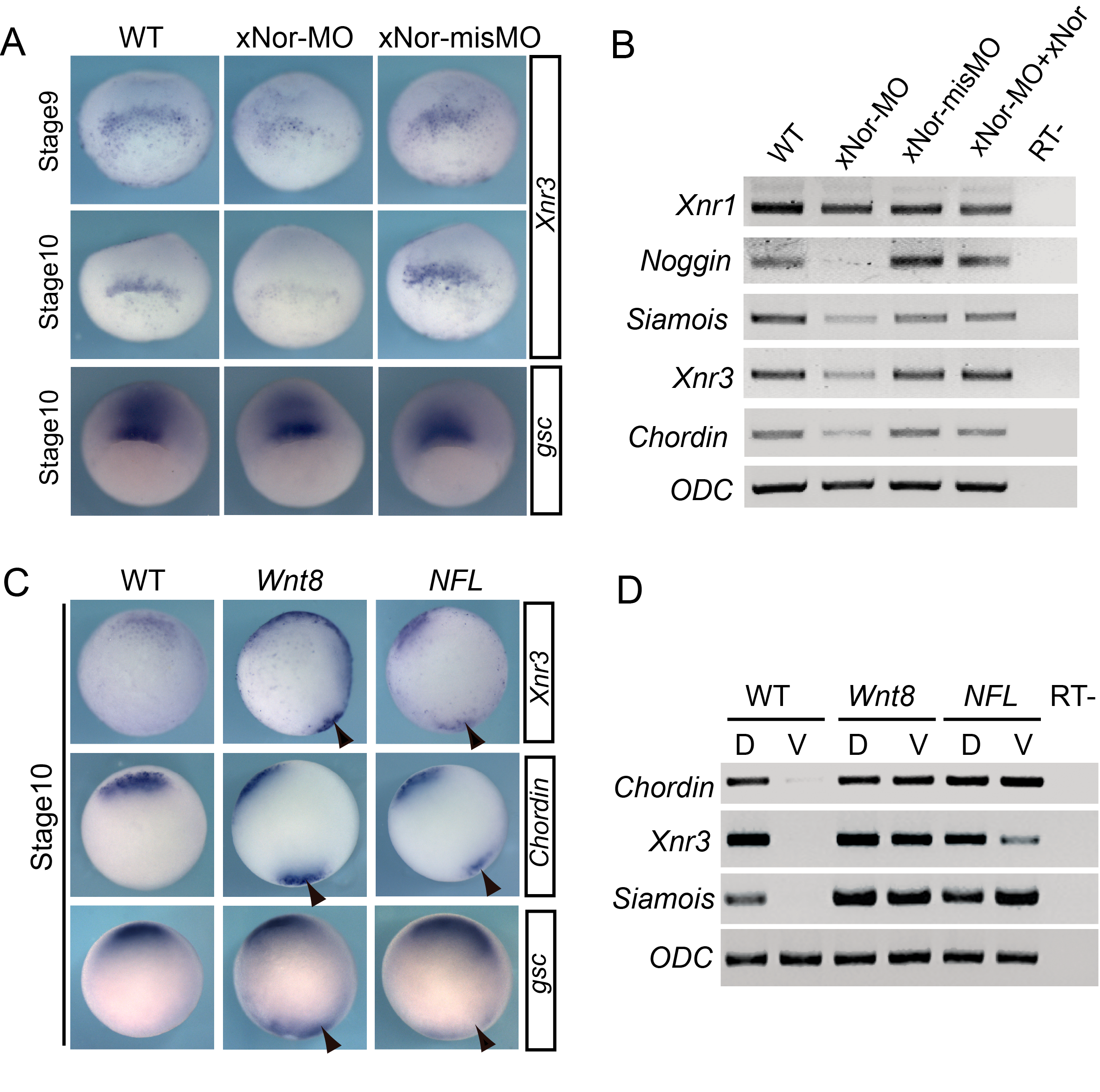

Supplement: Figure S4 — xNorrin is essential for early dorsal-specific gene expression. (A) The injection of xNor-MO reduced Xnr3 (stage 9: 70% reduced, n = 17; stage 10: 70% reduced, n = 20) but not gsc (14% reduced, n = 22) expression, as assayed by whole-mount in situ hybridization. (B) RT-PCR analysis showed that xNor-MO injected into dorsal animal cells reduced the expression of early dorsal-specific genes in stage 9 embryos. This reduction could be rescued by the injection of xNorrin mRNA (50 pg) lacking the xNor-MO target sequence. Note that xNor-MO did not change Xnr1 expression. ODC served as a loading control. (C) The overexpression of NFL in ventral vegetal blastomeres induced Wnt target gene expression. Upon injection into the ventral blastomeres of early eight-cell embryos, both NFL and Wnt8 induced Xnr3 (NFL: 56%, n = 25; Wnt8: 83%, n = 46) and Chordin (NFL: 72%, n = 29; Wnt8: 88%, n = 60) expression. However, NFL only weakly induced the Spemann organizer marker gsc (NFL: 7%, n = 28; Wnt8: 84%, n = 50). All embryos are in vegetal views. Arrowheads indicate the injection sites. (D) RT-PCR results (not quantitative) showed that NFL injection into ventral-vegetal cells ectopically activated Wnt target genes (Chordin, Siamois, and Xnr3) at the ventral side of the embryos. ODC served as a loading control. (TIF) [file pbio.1001286.s004.tif]

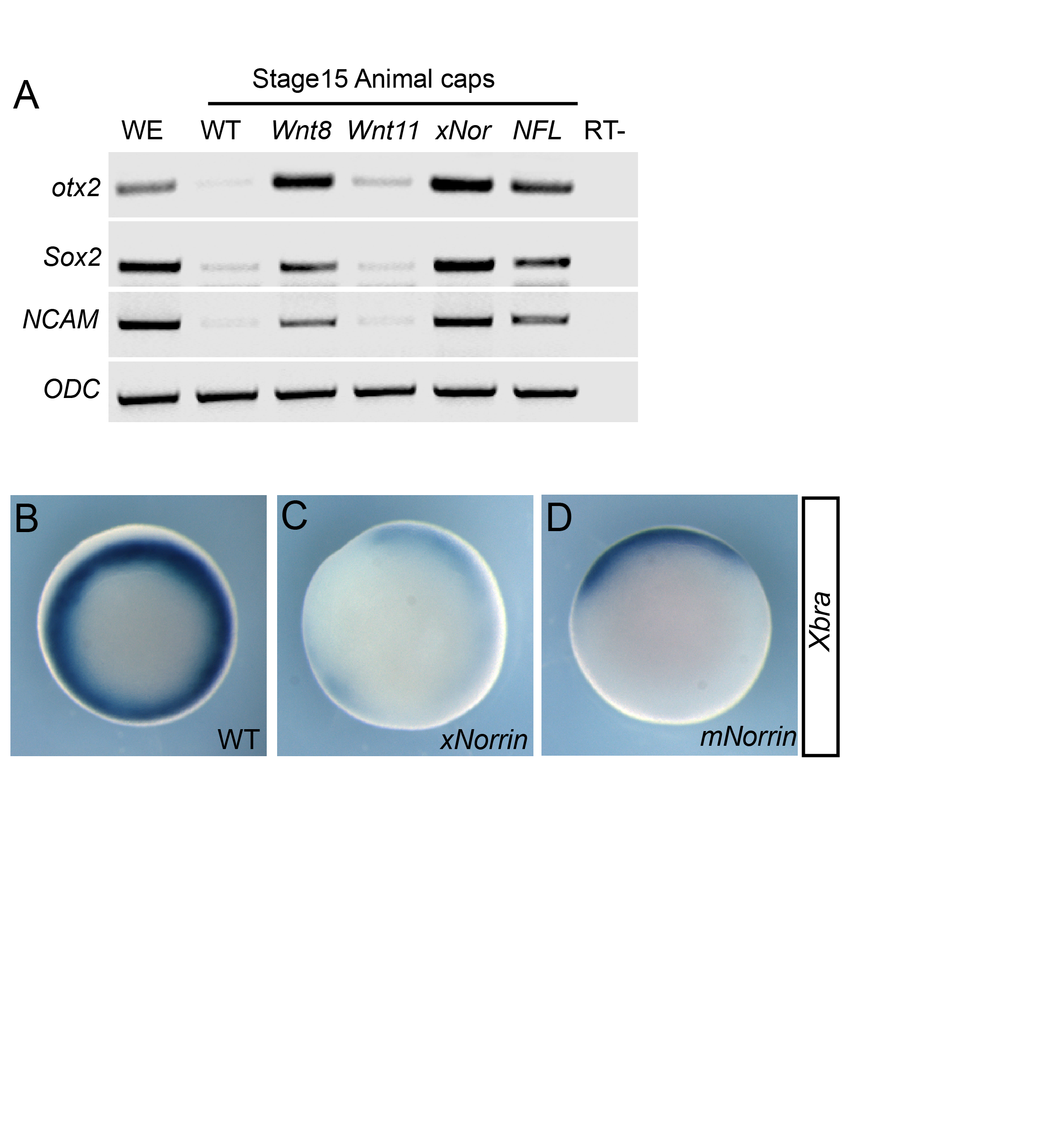

Supplement: Figure S5 — xNorrin induces neural formation and inhibits mesoderm formation. (A) RT-PCR analysis of neural gene expression in stage 15 animal caps from embryos injected with Wnt8 (20 pg), Wnt11 (200 pg), xNorrin (xNor) (200 pg), and NFL (200 pg each). The expression of otx2, Sox2, and NCAM (all neural markers) was analyzed. ODC served as a loading control. (B–D) Xbra expression detected in whole-mount in situ hybridization. A wild-type embryo at stage 10.5 (B); reduced Xbra expression in stage 10.5 embryos injected with xNorrin RNA (200 pg) at the vegetal pole at the two-cell stage (82% reduced, n = 34) (C); reduced Xbra expression in stage 10.5 embryos injected with mouse Norrin RNA (200 pg) at the vegetal pole at the two-cell stage (66% reduced, n = 36) (D). (TIF) [file pbio.1001286.s005.tif]

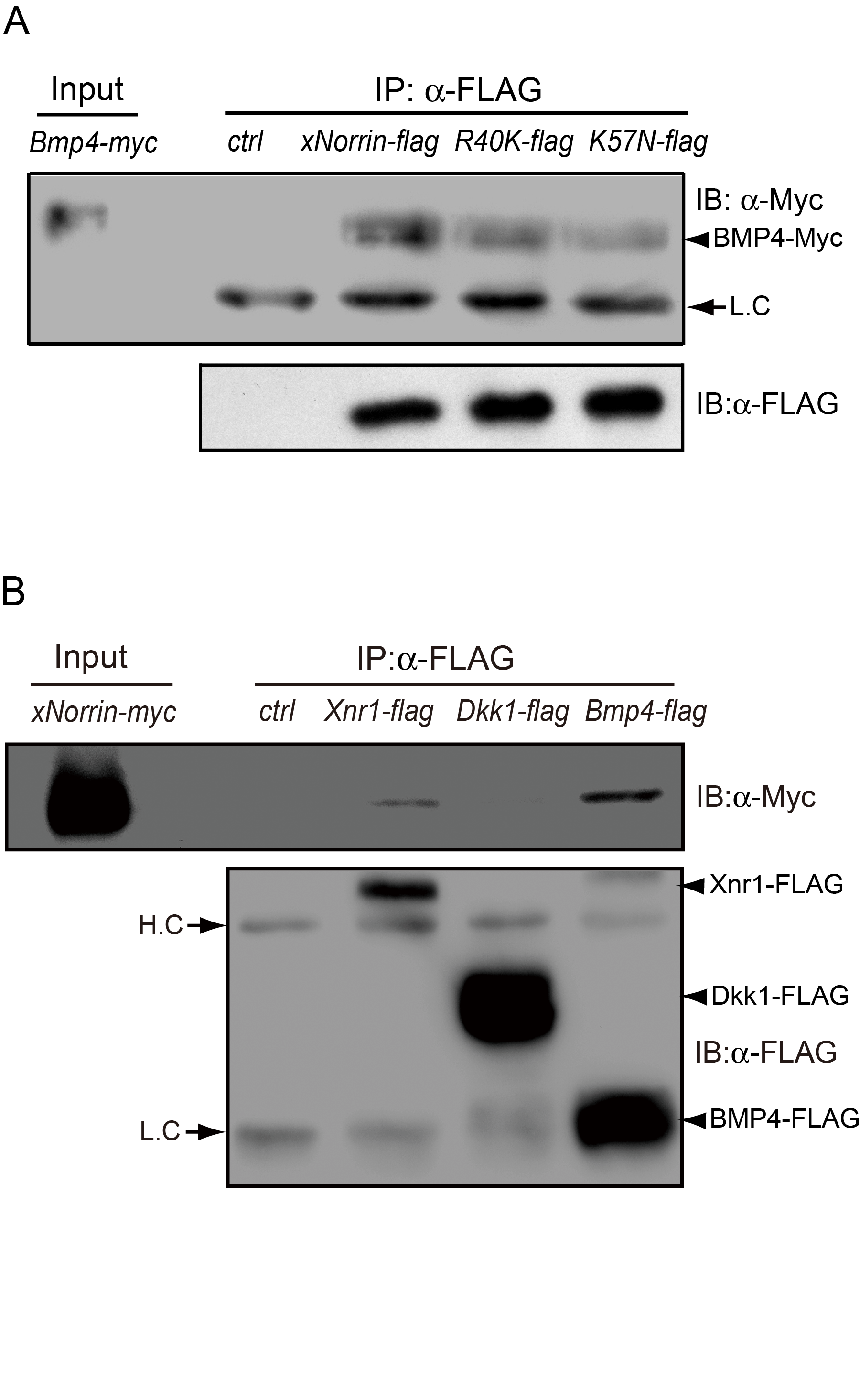

Supplement: Figure S6 — xNorrin interacts with TGF-β family members. (A) xNorrin binds to BMP4. Epitope-tagged wild-type xNorrin or xNorrin point mutants (R40K or K57N) and BMP4 were separately expressed in HEK293 cells. The conditioned medium from cells expressing individual xNorrin and BMP4 were mixed and incubated. The FLAG-tagged protein complexes were immunoprecipitated using an anti-FLAG antibody and separated in SDS-PAGE and blotted. An anti-c-Myc antibody was used to detect Myc-tagged BMP4. The expression of FLAG-tagged proteins was detected using an anti-FLAG antibody. xNorrin was shown to bind to BMP4. The R40K mutant retained this binding activity, while the K57N mutant showed slightly reduced BMP4 binding. Conditioned medium of the parent FLAG-plasmid-transfected cells was used as a control (ctrl). Arrowhead, BMP4-Myc; arrow, immunoglobulin light chains (LC). α-FLAG, anti-FLAG tag monoclonal antibody; α-Myc, anti-c-Myc-tag monoclonal antibody. (B) xNorrin binds to Xnr1 but not DKK-1. The assay performed was similar to that described in (A). Conditioned medium (ctrl) was the same as in (A). Arrowheads indicate FLAG-tagged protein. Arrows point to immunoglobulin heavy chain (HC, top) and light chain (LC, bottom). (TIF) [file pbio.1001286.s006.tif]

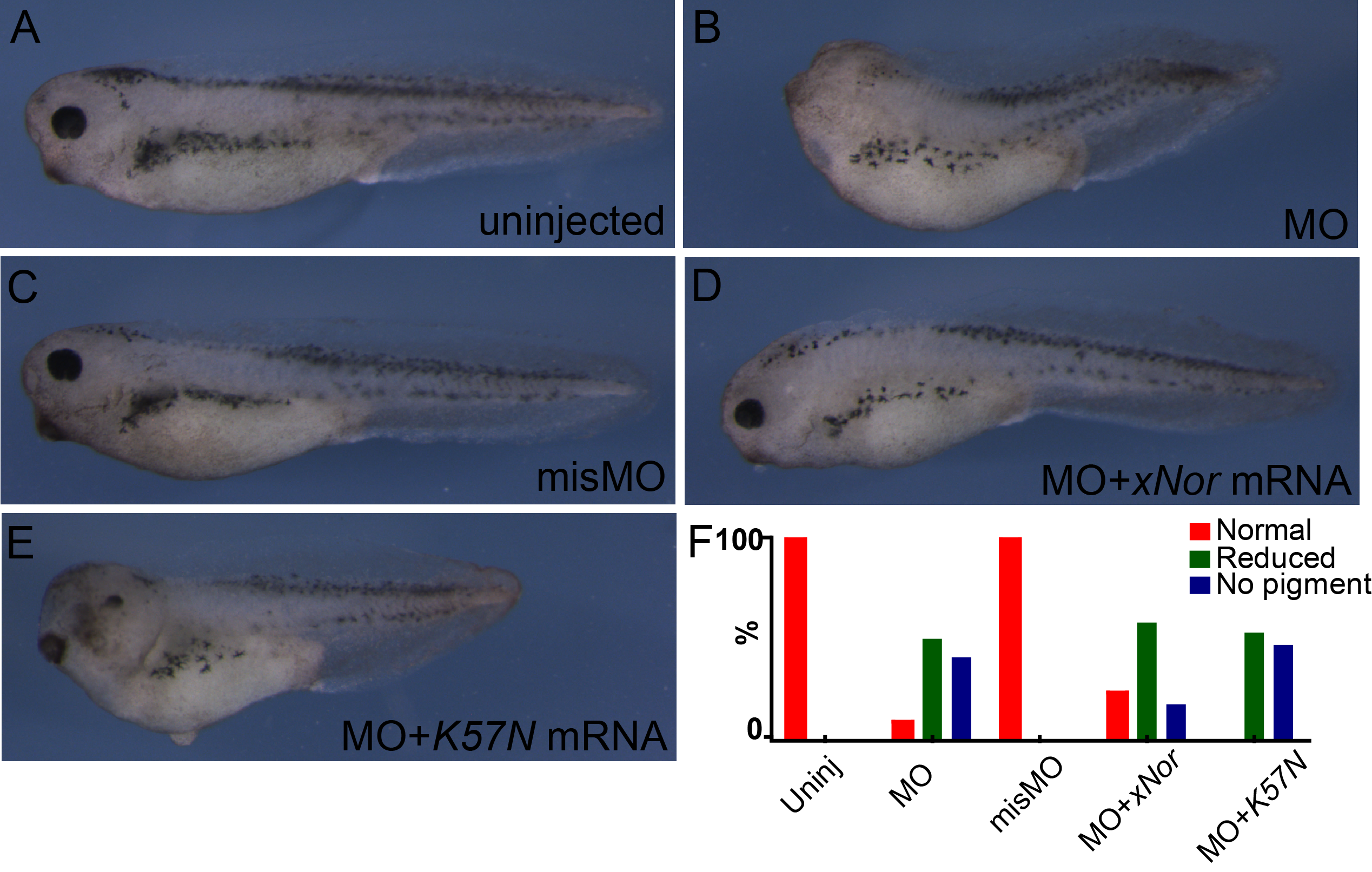

Supplement: Figure S7 — The xNorrin K57N mutant failed to efficiently rescue anterior defects in xNorrin morphants. (A–E) An uninjected embryo (A). A xNor-MO (20 ng)–injected embryo (B). Note the lack of eye pigment. A xNor-misMO (20 ng)–injected embryo (C). A xNor-MO and wild-type Norrin RNA (50 pg) co-injected embryo (D). A xNor-MO and xNorrin K57N RNA (50 pg) co-injected embryo (E). (F) Summary of anterior defect frequency in (A–E). Uninjected: n = 40; MO: n = 24; xNor rescue: n = 20; K57N rescue: n = 19; misMO: n = 15. RNAs and MO were injected into the dorsal animal region at the four-cell stage. All embryos shown are around stage 36. (TIF) [file pbio.1001286.s007.tif]
